# Aconine attenuates osteoclast-mediated bone resorption and ferroptosis to improve osteoporosis via inhibiting NF-κB signaling

**DOI:** 10.3389/fendo.2023.1234563

**Published:** 2023-11-13

**Authors:** Chunchun Xue, Huan Luo, Libo Wang, Qing Deng, Wenyun Kui, Weiwei Da, Lin Chen, Shuang Liu, Yongpeng Xue, Jiafan Yang, Lingxing Li, Wenlan Du, Qi Shi, Xiaofeng Li

**Affiliations:** ^1^ Shanghai Municipal Hospital of Traditional Chinese Medicine, Shanghai University of Traditional Chinese Medicine, Shanghai, China; ^2^ Department of Pharmacy, the Second Affiliated Hospital, Zhejiang University School of Medicine, Hangzhou, China; ^3^ Longhua Hospital, Shanghai University of Traditional Chinese Medicine, Shanghai, China

**Keywords:** aconine, osteoporosis, osteoclast, ferroptosis, NF-κB signaling

## Abstract

Osteoporosis (OP), a prevalent public health concern primarily caused by osteoclast-induced bone resorption, requires potential therapeutic interventions. Natural compounds show potential as therapeutics for postmenopausal OP. Emerging evidence from *in vitro* osteoclastogenesis assay suggests that aconine (AC) serves as an osteoclast differentiation regulator without causing cytotoxicity. However, the *in vivo* functions of AC in various OP models need clarification. To address this, we administered intraperitoneal injections of AC to ovariectomy (OVX)-induced OP mice for 8 weeks and found that AC effectively reversed the OP phenotype of OVX mice, leading to a reduction in vertebral bone loss and restoration of high bone turnover markers. Specifically, AC significantly suppressed osteoclastogenesis *in vivo* and *in vitro* by decreasing the expression of osteoclast-specific genes such as *NFATc1*, *c-Fos*, *Cathepsin K*, *and Mmp9*. Importantly, AC can regulate osteoclast ferroptosis by suppressing Gpx4 and upregulating Acsl4, which is achieved through inhibition of the phosphorylation of I-κB and p65 in the NF-κB signaling pathway. These findings suggest that AC is a potential therapeutic option for managing OP by suppressing NF-κB signaling-mediated osteoclast ferroptosis and formation.

## Introduction

Osteoporosis (OP), a common metabolic skeleton disorder characterized by low peak bone mass and increased susceptibility to fracture, is a major public healthy burden for elderly population and postmenopausal women (1). According to estimations, a 50-year-old white woman has 50% lifetime risk of osteoporotic fracture, while all postmenopausal women over the age of 65 have a history of fractures (2, 3). For healthy bone density, the organ of bone often undergoes continuous remodeling to control bone mass, which involves two essential processes: osteoclast-mediated bone resorption (breakdown) and osteoblast-mediated bone formation (build-up) (4). When the activity of osteoclasts is increased, osteoblastic bone formation does not keep pace with bone resorption, excessive bone resorption results in bone loss (5, 6). Therefore, compounds with anti-osteoclastogenic properties may represent promising therapeutic agents for osteoprotection.

Osteoclasts, derived from hematopoietic precursor cells in the bone marrow, whose differentiation and maturation are regulated by a variety of systemic cytokines and signaling (7, 8). Mature osteoclasts with multinuclear features can tightly attach to the bone surface, leading to bone resorption (9). During osteoclast differentiation, bone marrow-derived macrophages (BMMs) are elevated after nuclear factor(NF)-kappa B ligand (RANKL) stimulation, followed by NF-κB and MAPK signaling activation, and subsequently stimulate osteoclastogenesis by promoting the specific genes expression that typify the osteoclast lineage, including c-Fos, Mmp9, Cathepsin K and T cell nuclear factor cytoplasmic 1(NFATc1) (10, 11).

Growing evidence reveals that ferroptosis, a new form of cell death, contributes to several degenerative disorders, including age-related OP (12, 13). It features iron overload and accumulation of lipid peroxides, and the specific biological characteristics presents downregulation of glutathione peroxidase 4 (Gpx4) expression and upregulation of acyl-CoA synthetase long-chain family member 4 (Acsl4) (14). Emerging reports indicate that ferroptosis plays critical role in the development of RANKL-induced osteoclasts differentiation (15), and the higher level of ferroptosis, the stronger osteoclast activities, indicating that the potential benefit of regulating ferroptosis in osteoclast to prevent bone loss.

Aconine (AC), a diester alkaloid isolated from a traditional Chinese medicine *Aconiti Lateralis Radix Preparata* (Fuzi), has been proven to be safe for human consumption (16), It mainly functions to recess inflammation in arthritis and heart-protective effects (17, 18). Furthermore, another study has demonstrated that AC suppressed RANKL-induced osteoclast differentiation in RAW264.7 cells by inhibiting NF-κB and NFATc1 activation (19). However, whether AC regulates *in vivo* osteoclast formation and activity as well as OP progression has not yet been explored.

Therefore, in the present study, the efficacy of AC in preventing bone loss in ovariectomy (OVX)-induced OP mice, as well as the inhibitory effect on osteoclast activities *in vivo* and *in vitro* were investigated. Moreover, we sought to decipher the potential working mechanisms of AC on osteoclast ferroptosis through the NF-κB signaling pathway. These findings will provide comprehensive insight into the potential therapeutic implications and underlying mechanisms of AC for treating OP.

## Materials and methods

### Animals

Female C57/BL6 mice at the age of 8 weeks were purchased from Shanghai JSJ Laboratory Animal Co., Ltd. (Shanghai, China) and housed at the animal facility of Shanghai Municipal Hospital of Traditional Chinese Medicine (TCM) under standard conditions. 8-10-week-old male *Opg* knockout mice and C57/BL6 mice were purchased from the Shanghai Research Center of Model Organisms. All animal experiments were approved by the Animal Experiments Ethical Committee of Shanghai Municipal Hospital of TCM (No. 2022022).

### Ovariectomy -induced OP mouse model and treatments

Twenty-four mice were randomly divided into three groups (n = 6 per group): sham group, OVX group, and OVX + AC group. Bilateral ovariectomies were performed sodium pentobarbital (40mg/kg) anesthesia to induce OP in the OVX and OVX+AC groups. For the sham group, a portion of the adipose tissue surrounding the ovaries was removed without resecting them. On day 7 after the operation, mice in OVX + AC group were intraperitoneally injected with AC (5 mg/kg) once daily for 8 weeks, while the sham and OVX group mice were received a vehicle control injection.

### Micro-CT analysis

The L5 lumbar vertebra were harvested from the sacrificed mice. All data were acquired using a Skyscan 1172 μCT scanner (Bruker, Kontich, Belgium) at a source voltage of 49 kV and a source current of 179 uA with a voxel size of 10 μm. The region of interest (ROI) pertaining to the L5 lumbar vertebra can be described as a cylindrical volume. This volume is defined by a circular base with a radius of 2 mm, located at the bottom of the L5 vertebral body, and extending upwards for the height of the L5 vertebra. The parameters of ROI were analyzed as follows: bone mineral density (BMD), bone volume/tissue volume (BV/TV), trabecular thickness (Tb.Th), trabecular number (Tb.N), and trabecular separation (Tb.Sp), which were measured from 3D images of cancellous bone reconstructed from the bitmap dataset.

### Histology and staining

Samples were fixed in 10% neutral-buffered formalin, decalcified in ethylenediaminetetraacetic acid (EDTA) solution (pH 7.4), dehydrated, and embedded in paraffin. Serial midsagittal sections (4 μm thick) were then cut and stained with hematoxylin and eosin (H&E) staining for morphometric analysis.

The immunohistochemistry (IHC) staining was performed according to the manufacturer’s instructions for SP Link Detection Kits (ZSGB-BIO, PV-9001/PV9002) as we previously described (20). Briefly, paraffin sections were rehydrated and digested with proteinase K solution (10 mg/ml) for 20 minutes at 37°C. Then the sections were respectively incubated with primary antibodies of Runx2 (Abcam, ab192256, 1:400), Osterix (Abcam, ab 209484, 1:400), Mmp9 (Abcam, ab38898, 1:1000), Cathepsin K (Abcam, ab19027, 1:500), NFATc1 (Abcam, ab2796, 1:500), c-Fos (Abcam, ab222699, 1:500), Gpx4 (Abcam, ab125066, 1:1000), Acsl4 (Abcam, ab155282, 1:1000), p-p65 (CST, #3033, 1:500) and p-I-κB (CST, #2859, 1:500) overnight at 4°C. Followed by coloration with 3,3’-Diaminobenzidine (DAB) solution and hematoxylin counterstaining, dehydration, clearance, and mounting. The stained sections were captured with a light microscope (Leica, DM6).

For the immunofluorescence (IF) staining, the expression of p65 in the L5 lumbar vertebra was determined by IF staining as previously described (20). Paraffin sections (4 μm thick) of the lumbar vertebra among groups were treated with a primary antibody anti-rabbit p65 (CST, #8242, 1:1000) overnight at 4°C, then incubated with fluorescent-labeled secondary antibodies for 1 hour, and counterstained with DAPI. Finally, the sections were scanned using a microscope (Leica, DM6).

### Tartrate-resistant acid phosphatase staining

In this study, the L5 vertebra among groups was performed TRAP staining (Wako, #294-67001) according to the manufacturer’s recommendations. In brief, paraffin sections (4 μm thick) were rehydrated and applied with a sufficient TRAP staining solution for 30 minutes at room temperature (RT). Distilled water to soak the sections and sufficient of nuclear staining solution was applied for 4 ~ 5 seconds, then immediately wash one section by moving them up and down in distilled water. Dry the sections on a heater plate at 37°C and mounting them with xylene. In the middle of the L5 vertebral adjacent to the intervertebral disc, the number of osteoclasts to bone surface ratios (20x field) was quantified.

### Enzyme-linked immunosorbent assay

Serum markers of bone turnover were detected using the appropriate ELISA kits in strict accordance with the manufacturer’s instructions. The cleaved-off of type I collagen (PINP) (Sangon Biotech, D721053) and bone-specific alkaline phosphatase (BALP) (Sangon Biotech, D721049) were performed for bone formation, while carboxy-terminal cross-linking telopeptide of type I collagen (β-CTX) (Sangon Biotech, D721187) was detected for bone resorption.

Serum aspartate transaminase (AST), alanine transaminase (ALT) and blood urea nitrogen (BUN), creatinine (Cr) were detected by Laboratory Department of Longhua Hospital Affiliated to Shanghai University of Traditional Chinese Medicine.

### *In vitro* osteoclastogenesis assay

Bone marrow cells were isolated from 10-week-old WT or *Opg* KO mice by flushing the marrow space of femora and tibiae. The cells were then plated at a density of 8 × 10^3^ cells/mL in 96-well plates and treated with M-CSF (50 ng/mL) for 3-4 days to induce macrophage enrichment. After 3-4 days, the cells were treated with various concentrations of AC (0, 10, or 20 μM) followed by M-CSF (200 ng/mL) and RANKL (200 ng/mL) stimulation until osteoclasts differentiated in the control group. When fully mature multinucleated osteoclasts were detected, TRAP activities were performed using a TRAP assay kit (Solarbio, G1492). If TRAP-positive multinucleated cells had three or more nuclei, they were classified as osteoclast-like cells.

### RNA sequencing

To further explore the molecular mechanisms underlying AC treatment against osteoclast formation, we performed bioinformatics analysis of mRNA transcriptomes. BMMs from WT mice were seeded in a 12-well plate and cultured with osteoclast-stimulating medium (200 ng/mL M-CSF and 200 ng/mL RANKL) in the presence or absence of 20 μM AC subjected to 48 h incubations. Cells then were collected for RNA sequencing. Total RNA was isolated using the Trizol Reagent (Invitrogen Life Technologies), after which the concentration, quality and integrity were determined using a NanoDrop spectrophotometer (Thermo Scientific). Three micrograms of RNA were used as input material for the RNA sample preparations. Sequencing libraries were generated according to the following steps. Firstly, mRNA was purified from total RNA using poly-T oligo-attached magnetic beads. Fragmentation was carried out using divalent cations under elevated temperature in an Illumina proprietary fragmentation buffer. First strand cDNA was synthesized using random oligonucleotides and Super Script II. Second strand cDNA synthesis was subsequently performed using DNA Polymerase I and RNase H. Remaining overhangs were converted into blunt ends via exonuclease/polymerase activities and the enzymes were removed. After adenylation of the 3′ends of the DNA fragments, Illumina PE adapter oligonucleotides were ligated to prepare for hybridization. To select cDNA fragments of the preferred 400-500 bp in length, the library fragments were purified using the AMPure XP system (Beckman Coulter, Beverly, CA, USA). DNA fragments with ligated adaptor molecules on both ends were selectively enriched using Illumina PCR Primer Cocktail in a 15-cycle PCR reaction. Products were purified (AMPure XP system) and quantified using the Agilent high sensitivity DNA assay on a Bioanalyzer 2100 system (Agilent). The sequencing library was then sequenced on NovaSeq6000 platform (Illumina) Genekinder Medicaltech (Shanghai) Co., Ltd, China.

### RNA extraction and RT-PCR assay

BMMs from WT or *Opg* KO mice were seeded in a 24-well plate and cultured with osteoclast-stimulating medium (200 ng/mL M-CSF and 200 ng/mL RANKL) in the presence or absence of AC for 5 days to form osteoclasts. Cells were collected for examination into the expression of osteoclast-specific genes using RT-PCR. Total RNA was extracted using EZ-press RNA purification (EZBioscience, B0004D) and was reverse transcribed by 1 μg using the Reverse Transcription Kit (TaKaRa, RR037A). cDNA was amplified by RT-PCR using an SYBR Green qPCR Kit (TaKaRa, RR420A) with sequence-specific primers (**Supplemental Table 1**). Each sample was repeated with 3 independent RT-PCR amplifications. Fold changes of genes of interest were calculated using control samples as 1.

### Western blot

To determine the effect of AC on osteoclast function or ferroptosis levels, BMMs were seeded in 6 cm plates and cultured with osteoclast-stimulating medium in the presence or absence of 20 μM AC subjected to 48 h incubations. Total cellular proteins were collected to detect the osteoclast or ferroptosis-specific makers.

Protein levels were determined using a BCA protein assay kit (Beyotime) and Chemiluminescence reagent (Beyotime) was used to visualize protein bands. All data were acquired using the ChemiDOC Imaging System (BIO-RAD). The antibodies used for Western blot were Cathepsin K (Abcam, ab19027, 1:1000), c-Fos (CST, #2250, 1:1000), Gpx4 (Abcam, ab125066, 1:1000), Acsl4 (Abcam, ab155282, 1:1000), and GAPDH (Beyotime, A0208, 1:3000). GAPDH was used as an internal control.

To confirm the connection between NF‐κB signaling and osteoclast formation/ferroptosis. BMM cells were pretreated with 10 ng/mL recombinant NF-κB (Novoprotein, #CR72) for 2 h followed by osteoclast-stimulating medium in the presence or absence of 20 μM AC for 24 h. Total proteins were harvested to assess the changes in key markers associated with osteoclast or ferroptosis proteins.

### Statistics analysis

Data were presented as mean ± standard deviation (SD) and were analyzed using the GraphPad Prism software (4.0). One-way analysis of variance (*ANOVA*) followed by least significant difference (*LSD*) or Tamhane’s T2 was used for multiple comparisons. *P* values < 0.05 were considered statistically significant.

## Results

### AC prevents bone mass loss in OVX-induced OP mice

**Figure 1:**
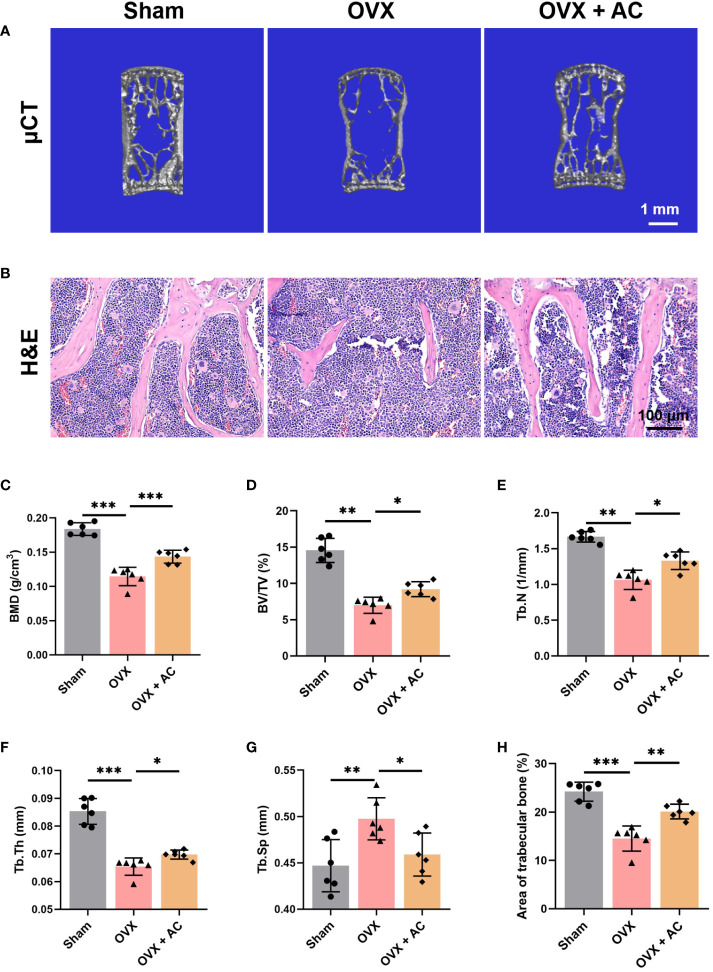
AC protects against bone loss in OVX-induced OP mice. **(A)** Representative μCT images of 3D of L5 lumbar vertebrae. Scale bar: 1 mm. **(B)** Representative images of H&E staining of L5 lumbar vertebrae in each group. Scale bar: 100 μm. Quantitative analyses of parameters regarding the bone architecture of L5 lumbar vertebrae, including **(C)** bone mass density (BMD), **(D)** bone volume/tissue volume (BV/TV), **(E)** trabecular thickness (Tb. Th), **(F)** trabecular number (Tb. N), and **(G)** trabecular separation (Tb. Sp). **(H)** The ratio of the area of trabecular bone. Data presented as means ± s.d. *P < 0.05, **P < 0.01, ***P < 0.001. n = 6.

To determine the therapeutic potential of AC on OP progression *in vivo*, mice were subjected to OVX modeling and subsequently treated with AC via intraperitoneal injection once a day for 8 weeks. The L5 vertebrae of OVX mice were harvested and subjected to evaluate bone microstructure by μCT. The results of μCT showed that OVX mice exhibited obvious reductions in trabecular bone mass compared with the sham group, while AC treatment could effectively prevent bone loss in OVX mice (**Figure 1A**). Similarly, OVX mice demonstrated significantly decrease in BMD in the L5 vertebrae, versus the sham group (0.184 ± 0.009, vs. 0.115 ± 0.013, respectively; *P*<0.001), whereas AC therapy enhanced BMD (0.143 ± 0.009) accompanied by improved bone microstructural parameters, as indicated by increases in BV/TV, Tb.Th and Tb.N, and a decrease in Tb.Sp of the OVX mice (**Figures 1C–G**). Subsequently, histological examination using H&E staining was conducted in order to provide a more comprehensive understanding of the pathological alterations occurring in the bone structure. As expected, the trabecular bone of the OVX mice in L5 vertebrae exhibited a decrease in thickness, quantity, and areas. However, the administration of AC markedly ameliorated these alterations (**Figures 1B, H**). These data suggest that AC treatment has the potential to effectively prevent extensive bone loss in OP modeling.

### AC maintains bone homeostasis by blocking the high bone turnover and remodeling the OVX-induced osteoporotic microenvironment

**Figure 2:**
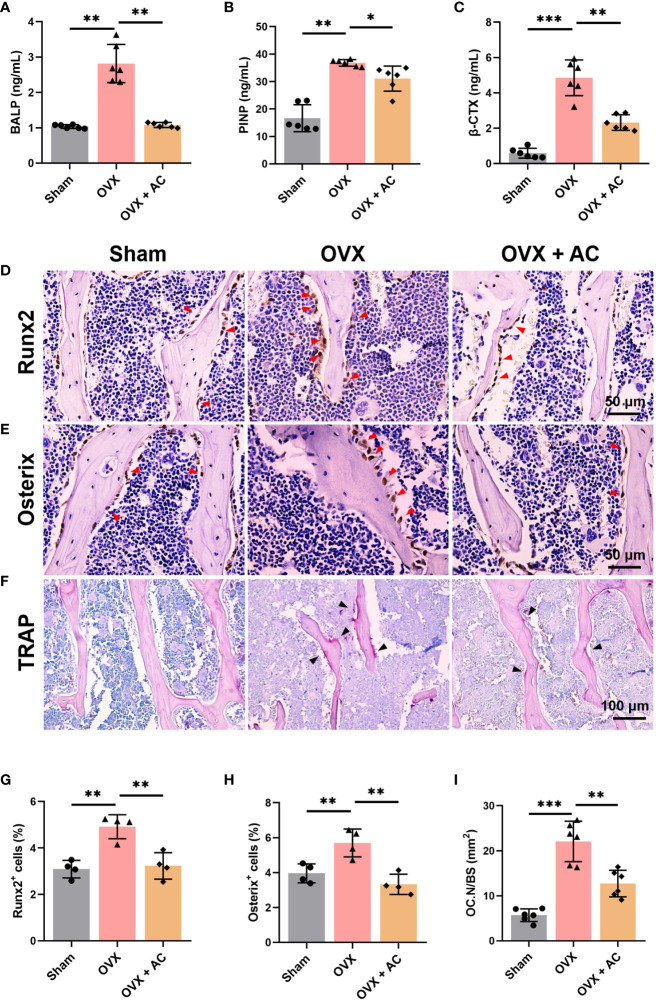
AC reverses the high bone turnover makers and remodels the osteoporotic pathophysiological microenvironment in OVX mice. Serum levels of bone formation markers of **(A)** bone-specific alkaline (BALP) and **(B)** N-terminal propeptide of type I collagen (PINP). **(C)** Serum β-CTX levels of the bone resorption marker. n = 6. IHC staining of **(D)** Runx2 and **(E)** Osterix in the lumbar vertebrae of each group. The red arrows represented the positive expression. **(F)** Representative of TRAP staining among groups. The black arrows represented the TRAP-positive cells. **(G, H)** Quantification of Runx2^+^ and Osterix^+^ cells. n = 4. **(I)** The ratio of the number of osteoclasts to per unit bone surface (OC.N/BS). n = 6. Data presented as means ± s.d. *P < 0.05, **P < 0.01, ***P < 0.001.

The maintenance of healthy bone mass is a multifaceted process that is governed by the removal of mineralized bone by osteoclasts and the subsequent replacement of new bone by osteoblasts (21, 22). Measurement of serum β-CTX levels present the degree of bone resorption, while serum PINP and BALP levels provide evidence of bone formation (23–25). Postmenopausal women with OP frequently have accelerated bone turnover activity, characterised by elevated serum levels of osteogenic and osteoclastic metabolic markers (26). Our study revealed that mice with OP produced by OVX displayed elevated levels of bone formation markers, including PINP and BALP, as well as the bone resorption marker β-CTX. However, these effects were greatly attenuated following injection of AC therapy (**Figures 2A–C**). Then, we conducted an IHC experiment to assess the expression of osteogenic-related proteins, Runx2 and Osterix. Notably, the results of IHC staining showed that the number of Runx2-expressing cells and Osterix-expressing cells located on the trabecular bone surface arranged in a shuttle shape was drastically increased in the OVX group relative to that in the sham group, the treatment of AC could reduce the excessive expression of Runx2 and Osterix (**Figures 2D, E, G**, **H**). In order to evaluate the effects of AC on osteoblast differentiation, we employed osteoblastic MC3T3-E1 cell line induced osteogenic induction media for 21 days to analyze ALP activities and mineralization ability by ALP staining and Alizarin Red-s (AR-S) staining. However, AC treatment showed no obvious inhibition of ALP activities and mineralization ability, compared to the DMSO group without AC treatment (**Supplemental Figure 1**). Next, TRAP staining was employed to evaluate the osteoclastogenesis. The results revealed that OVX modeling caused a notable increase in positive osteoclast staining within the trabecular bone of L5 vertebrae. Furthermore, the number of osteoclasts within the trabecular bone area were significantly increased by ~30% compared to the sham group, whereas AC has the ability to impede the osteoclastogenesis with reduction by ~50% of osteoclasts number in the trabecular bone area in OVX mice (**Figures 2F, I**). Thus, AC administration reduces the formation of osteoclasts and inhibits osteoblast related key regulators, as well as reinstates a state of elevated bone turnover in mice subjected to OVX operation. Importantly, the inhibitory effect of AC on osteoclastogenesis may be more pronounced than its anti-osteogenesis effect, hence providing a protective mechanism against bone loss.

### AC represses osteoclast-specific gene expressions

**Figure 3:**
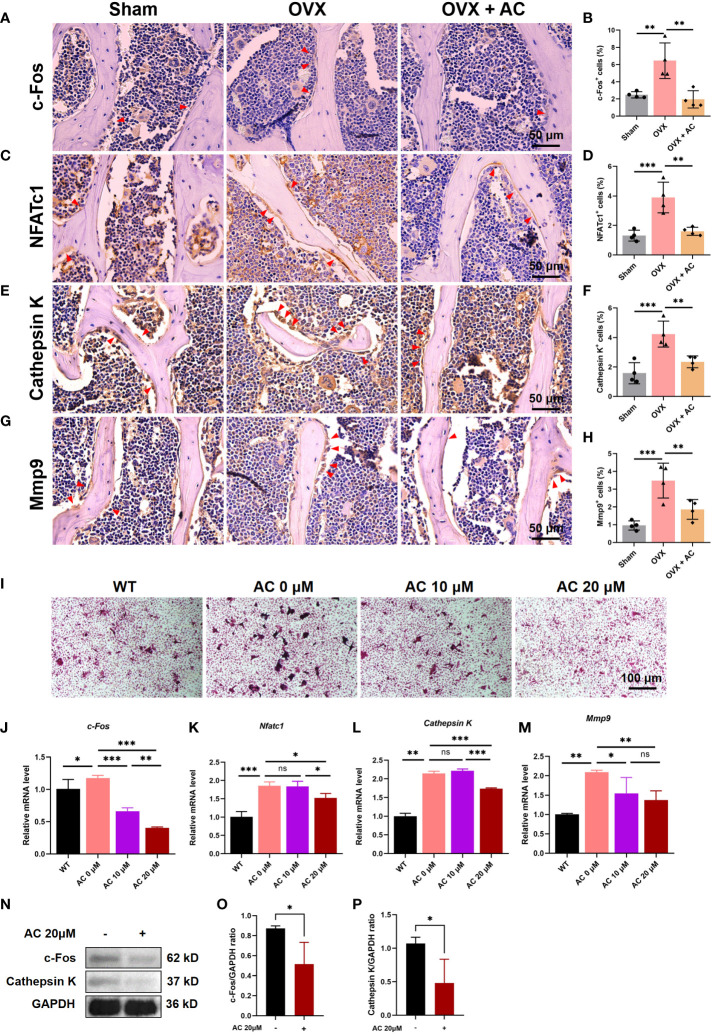
AC inhibits osteoclast formation and represses the expressions of osteoclast-specific genes. **(A–H)** IHC staining and quantitative analysis of c-Fos, NFATc1, Cathepsin K, and Mmp9 in the lumbar vertebrae among groups. Scale bar: 50 μm. n = 4. **(I)** BMMs were isolated from WT or *Opg* KO mice and were treated with different concentrations of AC (0, 10, or 20 μM) followed by osteoclast-inducing media with the M-CSF (200 ng/mL) and RANKL (200 ng/mL) stimulation until the osteoclasts differentiated in the control group. The osteoclast formation among groups was detected by TRAP staining. n = 3. **(J–M)** Expression levels of the osteoclastic-specific gene of *c-Fos*, *Nfatc1*, *Cathepsin K*, and *Mmp9*, respectively. Data presented as means ± s.d. *P < 0.05, **P < 0.01, ***P < 0.001. n = 3. **(N)** BMMs were isolated from WT mouse tibiae and femora and were cultured with osteoclast-stimulating medium (200 ng/mL M-CSF and 200 ng/mL RANKL) to stimulate osteoclast formation in the presence or absence of 20 μM AC subjected to 48 h incubations. The protein expression levels of c-Fos and Cathepsin K were determined by Western blot. GAPDH was used as an internal control. **(O)** The levels of c-Fos protein were normalized to that of GAPDH. **(P)** The levels of Cathepsin K protein were normalized to that of GAPDH.

Postmenopausal OP is caused by osteoclast-mediated bone resorbed surpassing osteoblast-mediated bone formed, leading to an imbalance in bone remodeling (27). Thus, we further assessed the changes in osteoclast function by determining the expression levels of osteoclast differentiation markers, including c-Fos, NFATc1, Cathepsin K, and Mmp9, using IHC staining. We found that OVX modeling upregulates the expression of c-Fos, NFATc1, Cathepsin K, and Mmp9 in the L5 vertebrae, and these increases were partially suppressed by treating OVX mice with AC (**Figures 3A–H**). There is evidence that osteoprotegerin (OPG) acts as a negative regulator of osteoclast formation by competing for the binding between the receptor activator of NF-κB (RANK) and its ligand (RANKL) (28, 29). Our and others’ research found that knockout of *Opg* resulted in severe OP phenotype with higher RANKL and osteoclast generation (30, 31). Thus, we cultured the bone marrow cells from *Opg* KO mice treated with different AC (0, 10 or 20 μM) to observe the effect of AC on osteoclast formation. Expectedly, AC treatment markedly inhibited osteoclastogenesis in a dose-dependent manner (**Figure 3I**), and AC at a high dose (20 μM) effectively downregulate their overexpression of *c-Fos*, *Nfatc1*, *Cathepsin K* and *Mmp*9 in osteoclasts (**Figures 3J–M**). To further investigate the effect of AC on osteoclast formation, BMMs were isolated from WT mouse tibiae and femora and were cultured with osteoclast-stimulating medium in the presence or absence of 20 μM AC subjected to 48 h incubations. As expected, AC demonstrated a significant inhibitory effect on the key regulators of osteoclast formation such as c-Fos and Cathepsin K (**Figures 3N–P**). Moreover, to exclude the possibility that the observed inhibitory effect of AC on osteoclastogenesis might be due to cytotoxicity, the viability of mouse leukemic monocyte/macrophage cell line RAW264.7 cells were treated with or without AC concentrations of 10 and 20 μM for 24 h. The CCK assay results revealed no significant variations in cell activity between AC 0, 10 and 20 μM (**Supplemental Figure 2**), demonstrating that AC had no obvious cytotoxic effect on osteoclast precursor cells at the concentrations used in this study. These findings reveal that AC treatment inhibits the key regulators of osteoclastogenesis, hence preventing excessive bone absorption.

### AC inhibits ferroptosis of osteoclasts in OVX mice

**Figure 4:**
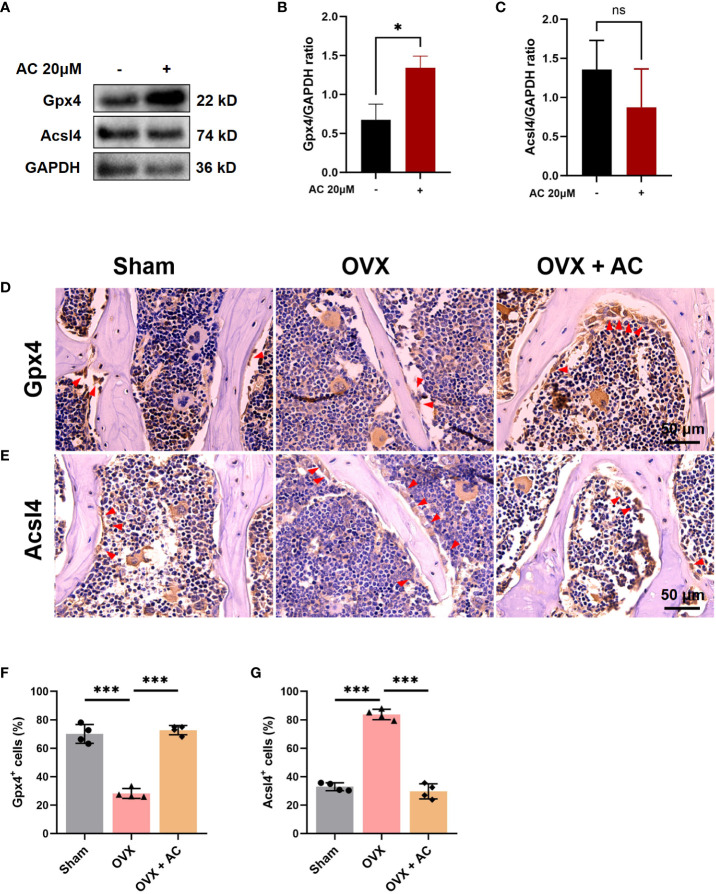
AC reverses the osteoclast ferroptosis in OVX mice. **(A)** BMMs, isolated from WT, were cultured with osteoclast-stimulating medium (200 ng/mL M-CSF and 200 ng/mL RANKL) to induce osteoclast formation in the presence or absence of 20 μM AC subjected to 48 h incubations. The protein expression levels of Gpx4 and Acsl4 were determined by Western blot. GAPDH was used as an internal control. **(B)** The levels of Gpx4 protein were normalized to that of GAPDH. **(C)** The levels of Acsl4 protein were normalized to that of GAPDH. **(D)** IHC staining of Gpx4 in the lumbar vertebrae of each group. Scale bar: 50 μm. **(E)** IHC staining of Acsl4 in the lumbar vertebrae among groups. Scale bar: 50 μm. **(F)** Quantitative analysis of Gpx4-positive cells in each group. **(G)** Quantitative analysis of Acsl4-positive cells in each group. The red arrows represented the positive cells. Data presented as means ± s.d. *P < 0.05, ***P < 0.001. n = 4.

Emerging evidence has demonstrated that ferroptosis was involved in RANKL-mediated osteoclastogenesis *in vitro* and *in vivo* (15). Therefore, we evaluated the effect of AC on osteoclast ferroptosis by detecting the key anti-ferroptosis protein, Gpx4, and a critical pro-ferroptosis marker, Acsl4. As determined *in vitro*, AC exhibited a promotive effect on the expression of critical anti-ferroptosis factors Gpx4, while concurrently suppressing the expression of the pro-ferroptosis protein Acsl4 in osteoclast (**Figures 4A–C**). Consistent with the effect of AC against ferroptosis-related key factors observed in BMMs, IHC staining showed that AC reversed the decreased expression of Gpx4 and enhanced expression of Acsl4 of osteoclasts in OVX mice (**Figures 4D–G**). These data imply that AC may have an effect on regulation for key genes of ferroptosis in osteoclast.

### AC suppresses the activation of the NF-κB signaling pathway in osteoclasts

**Figure 5:**
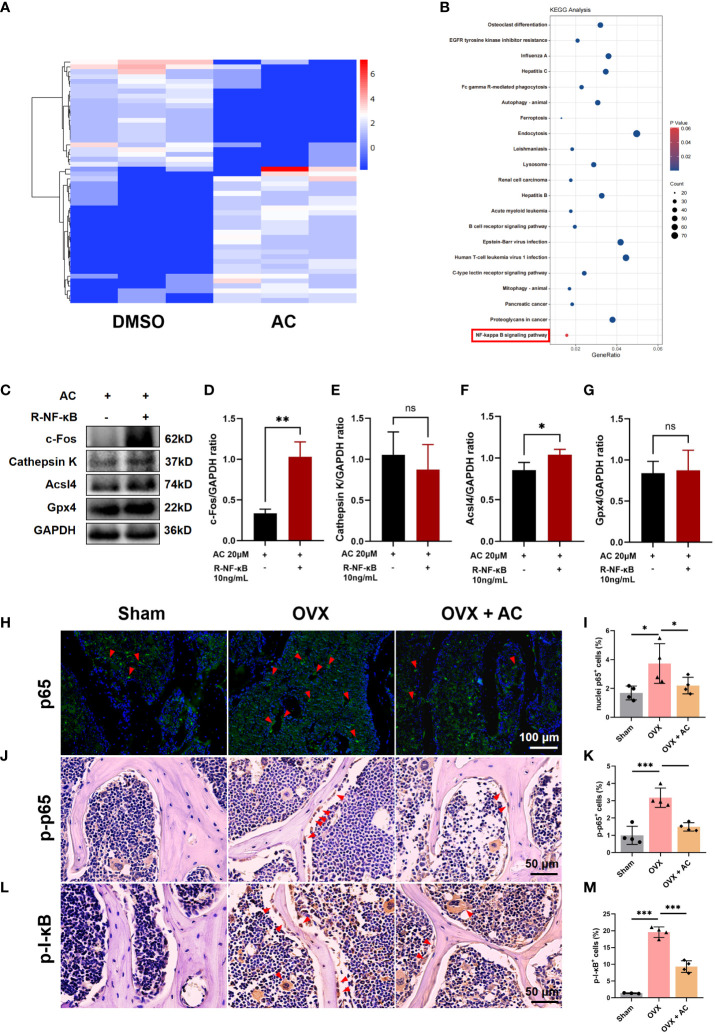
AC suppresses the NF-κB signaling pathway in osteoclasts. **(A)** The top 50 differentially expressed genes within up-and-down gene expressions were presented by heatmap analysis. **(B)** The KEGG analysis of differentially expressed genes between the AC and DMSO groups. **(C)** BMM cells were pretreated with 10 ng/mL recombinant NF-κB for 2 h followed by osteoclast-stimulating medium in the presence of 20 μM AC for another 24 h. Effect of recombinant NF-κB on expression of c-Fos, Cathepsin K, Acsl4 and Gpx4 proteins in AC-treated cells. GAPDH was used as an internal control. **(D–G)** Quantification of the expression levels of c-Fos, Cathepsin K, Acsl4 and Gpx4 proteins in **(C)**. **(H)** p65 nuclei translocation was presented by IF staining. Scale bar: 100 μm. **(I)** The relative percentage of p65 nuclei-positively stained cells to the total number of cells. **(J)** The expression of p-p65 in the lumbar vertebrae of each group. Scale bar: 50 μm. **(K)** Quantification of p-p65^+^ cells in each group. **(L)** The expression of p-I-κB in the lumbar vertebrae of each group. Scale bar: 50 μm. **(M)** Quantitative analysis of p-I-κB^+^ cells in each group. The red arrows represented the positive cells. Data presented as means ± s.d. *P < 0.05, **P < 0.01, ***P < 0.001. n = 3 - 4.

It has been reported that osteoclast formation and function are dependent on the NF-κB signaling pathways (32). To further explore the molecular mechanisms underlying AC treatment against osteoclast formation, we performed bioinformatics analysis of mRNA transcriptomes. As shown in **Figure 5A**, the heatmap illustrated the top 50 differentially expressed genes within up-and-down gene expressions in the AC group as compared to the DMSO group. KEGG analysis found that NF-κB signaling pathway were enriched in terms of differentially expressed genes within AC and DMSO groups (**Figure 5B**). To confirm the effect of NF‐κB signaling on osteoclast formation or ferroptosis subsequent to AC therapy, BMM cells were pretreated with 10 ng/mL recombinant NF-κB for 2 h followed by osteoclast-stimulating medium in the presence of 20 μM AC for another 24 h. We found that recombinant NF-κB protein treatment not only reversed the effect of AC on the expression of Gpx4 and Acsl4, but also increased the c-Fos expression, which had been inhibited by AC treatment (**Figures 5C–G**). Next, to gain a more comprehensive understanding of AC on regulation of NF-κB signaling transduction, the expression of p65 nuclei translocation in the L5 lumbar vertebra of each group was detected by IF staining. It can be seen that the nuclei levels of p65 were markedly elevated in OVX mice, however, the upregulation of p65 levels could be well inhibited by AC (**Figures 5H, I**). We then further investigated the expression of p-p65 and p-I-κB in L5 vertebrae using IHC staining. The results showed that the expression of p-p65 and p-I-κB in the osteoclast of OVX mice were both increased as compared to the sham group, whereas AC-treated OVX mice exhibited a decrease in the levels of p-p65 and p-I-κB (**Figures 5J–M**). Collectively, these results suggest that the inhibitory effects of AC on osteoclast formation and osteoclast-mediated ferroptosis are dependent on the activity of NF-κB signaling.

### AC treatment present a good biosafety for OVX-induced OP modeling

**Figure 6:**
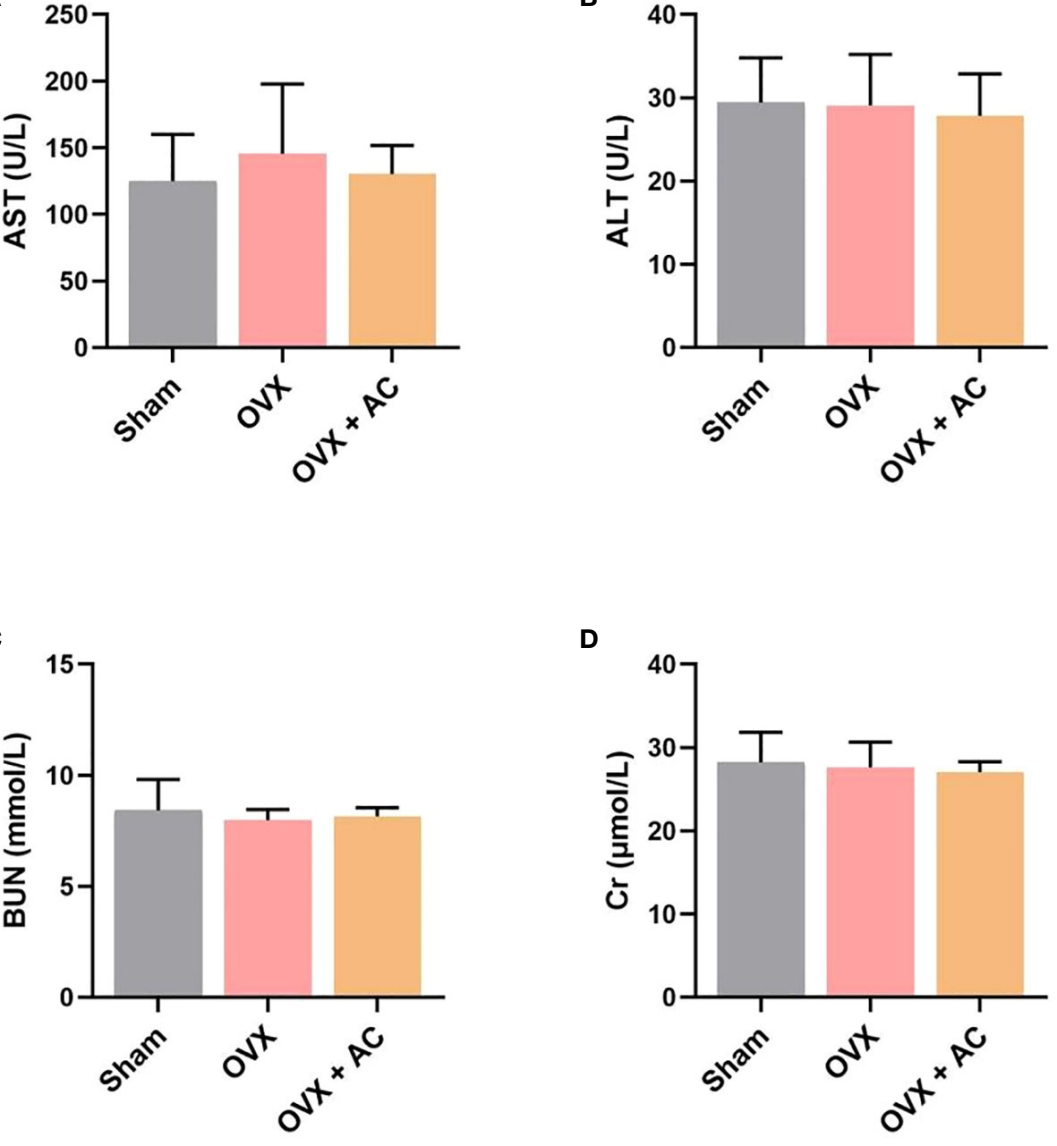
Effect of AC on chronic toxicity in OVX mice. Evaluation of the hepatotoxicity and nephrotoxicity of AC treatment by measuring the serum levels of **(A)** AST, **(B)** ALT, **(C)** BUN and **(D)** Cr. Data presented as means ± s.d., n = 4 - 6.

At the endpoint, the *in vivo* toxicity of AC was investigated by measuring the levels of hepatotoxicity markers (AST, ALT) and nephrotoxicity markers (BUN, Cr) in each group. Relative to the sham group, there was no discernible changes in the serum of AST, ALT levels and BUN, Cr levels after treatment with AC versus the OVX without AC treatment group (**Figures 6A–D**). Therefore, AC treatment presents a good biosafety for OP therapy.

## Discussion

**Figure 7:**
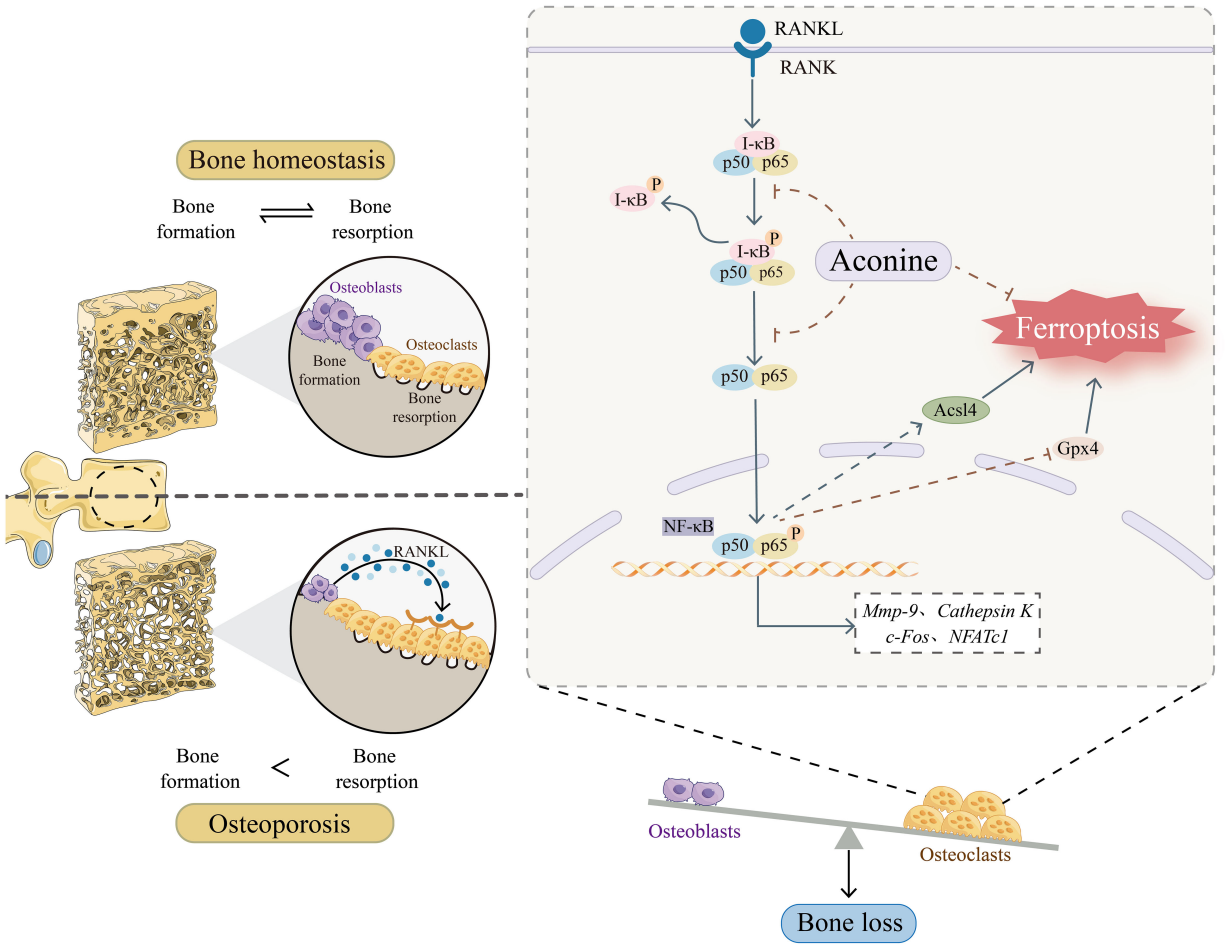
Schematic representation of the mechanism of AC treatment in OP.

During the development of OP, the activation of osteoclasts has the capacity to induce excessive bone resorption, leading to the deterioration of bone density and alterations in the microarchitecture of bone (33). Therefore, targeting the inhibition of osteoclasts activity is a promising therapeutic approach for addressing OP. AC, a non-toxic component of *Aconiti Lateralis Radix Preparata*, has been found to inhibit the RANKL-induced osteoclast formation and function in pre-osteoclastic RAW264.7 cells (19). However, there is limited knowledge regarding the impact of AC on bone loss and OP progression *in vivo*. In this study, we have successfully shown, for the first time, AC could reduce bone loss and inhibit the elevation of bone turnover markers in mice subjected to ovariectomy. Additionally, AC treatment was found to effectively lower both the numbers and activity of osteoclasts. Specifically, AC could prevent ferroptosis in osteoclasts by increasing Gpx4 and decreasing Ascl4, which is mediated by the inhibition of NF-κB activity (**Figure 7**). These findings elucidate a new potential perspective to the understanding of the mechanisms by which AC contributes to preventing bone loss in OP modeling.

During the menopausal phase, there is a notable rise in bone turnover and an expedited decline in bone density, resulting in an average loss of 11% in spinal bone density over the course of 10 years following menopause (34). Accumulating evidence has proven that high level of bone turnover correlates with increased biochemical indicators of both bone formation and bone resorption (35, 36). BALP, a well-established and dynamic marker of osteoblasts, plays a crucial role in bone formation and serves as a reliable measure of osteocyte formation and activity levels (37). The concentrations of serum PINP serve as an indicator of the cumulative quantity of new bone production in the skeletal system (38). β-CTX is a metabolic marker for representing osteoblast activity (34). Our result showed that the L5 vertebrae of OVX mice experienced a significant reduction in spinal bone mass, amounting to a loss of nearly 40%. Additionally, we observed elevated levels of BALP and PINP as well as an increased concentration of β-CTX in serum. suggesting a state of heightened bone turnover in OVX mice. In line with the findings of bone metabolic markers, OVX mice was shown that the expression levels of Runx2 and Osterix, which are key transcription factors involved in the differentiation of osteoblasts, were found to be elevated and meanwhile an increase in osteoclasts number was detected through TRAP staining. However, AC effectively restored bone loss, reduced rapid bone turnover and reversed the upregulated state of both bone formation and bone resorption. These observations collectively suggest that AC could regulate bone homeostasis by remodeling the OVX-induced osteoporotic microenvironment.

It is hypothesised that the inhibitory effect of AC on osteoclast function may outweigh its impact on bone production, resulting in protection against bone loss. Prior studies have revealed that OVX mice exhibit an abnormally high expression of osteoclasts-specific markers, such as NFATc1, c-Fos, Mmp9, and Cathepsin K (39, 40), Furthermore, the increased expression of genes linked with osteoclasts relies on the activation of NF-κB signaling (41–43). c-Fos is a critical factor for NFATc1 activation, and NFATc1 functions as the most transcription factor responsible for regulating the expression of osteoclast-specific genes, including Mmp9 and Cathepsin K (44). As expected, AC not only decreased NF-κB activity but attenuated the overexpression of NFATc1, c-Fos, Mmp9, and Cathepsin K in OVX mice. Additionally, the *in vitro* experiments showed a substantial anti-osteoclast effect in AC. These findings suggest that AC may effectively inhibit osteoclast activity by inactivating NF-κB signaling.

The NF-κB signaling pathway is involved in regulating several pathogenic processes (45, 46). Growing investigations have found that NF-κB signaling can modulate Gpx4-mediated ferroptosis in tumor cells (47, 48). Ferroptosis, a novel type of cell death mainly characterized by decreased activities of Gpx4 and cellular lipids composition by positive regulation of Acsl4 (49, 50), suggesting promoting Gpx4 or inhibition of Acsl4 may prevent ferroptosis (51). A current study used a RANKL-stimulated osteoclastogenesis assay to reveal that ferroptosis was involved in osteoclasts over the course of RANKL-induced differentiation (15). However, whether NF-κB signaling is associated with ferroptosis in osteoclast is largely elusive. Our results indicate that during the process of M-CSF/RANKL-induced osteoclastogenesis, the administration of AC treatment not only effectively suppressed the upregulation of c-Fos and Cathepsin K, which are crucial regulators of osteoclast formation, but also restored the abnormal expression of Gpx4 and Acsl4, two vital proteins involved in ferroptosis. These observed effects on inhibiting osteoclastogenesis and ferroptosis were found to be dependent on the activity of NF-κB signaling. These results suggest that NF-κB signaling pathway may play a critical role in regulating ferroptosis in osteoclasts and inhibition of NF-κB activity to modify osteoclast-mediated ferroptosis could be a valuable approach in the treatment of OP.

Besides, our study has certain limitations. *In vivo* experiments, we exclusively employed a singular dose concentration of AC, hence resulting in a dearth of comparative analysis including different concentrations of AC. Moreover, this study primarily identified the principal regulators of Gpx4 and Acsl4 in relation to ferroptosis, other the molecular alterations associated with AC targeting in the context of ferroptosis are needed for further investigation. Hence, in further study, we will set concentration gradients to treating OP models and perform *in vitro* osteoclastogenesis assay to further explore the ferroptosis phenotype in osteoclasts, this will enable us to enhance the logical understanding of anti-osteoclast and anti-ferroptosis in osteoclasts mediated by AC in the treatment of OP.

## Conclusion

In conclusion, our study demonstrated that AC, a natural product, could block the high concentration of bone turnover markers and remodel the OVX-induced osteoporotic microenvironment by inhibiting osteoclast formation and bone resorption function, thereby ameliorating OVX-induced OP phenotype in mice. Moreover, AC inhibited osteoclast ferroptosis by regulating the Gpx4 and Acsl4 expression via suppressing NF-κB signaling. These findings suggest that AC has the anti-resorptive and anti-ferroptosis properties, making it an excellent potential treatment option for postmenopausal OP.

## Data availability statement

The original contributions presented in the study are included in the article/supplementary materials, further inquiries can be directed to the corresponding author/s.

## Ethics statement

The animal study was approved by Animal Experiments Ethical Committee of Shanghai Municipal Hospital of TCM. The study was conducted in accordance with the local legislation and institutional requirements.

## Author contributions

XL conceived and designed the experiments. CX and HL performed the experiments and drafted the manuscript. LW performed the *in vitro* experiments and analyzed the data. QD, WK, WWD, LC, SL, YX, JY. LL, and WLD contributed to data collection. WK drafted the scheme of this study. XL takes responsibility for the integrity of the data analysis. QS supervised the work. All authors approved the final manuscript. All authors contributed to the article.
