# Supplementary material for: Aconine attenuates osteoclast-mediated bone resorption and ferroptosis to improve osteoporosis via inhibiting NF-κB signaling
Source: Front Endocrinol (Lausanne). 2023 Nov 13;14:1234563. doi: 10.3389/fendo.2023.1234563 (PMC10682992; doi:10.3389/fendo.2023.1234563)

Figure 1A

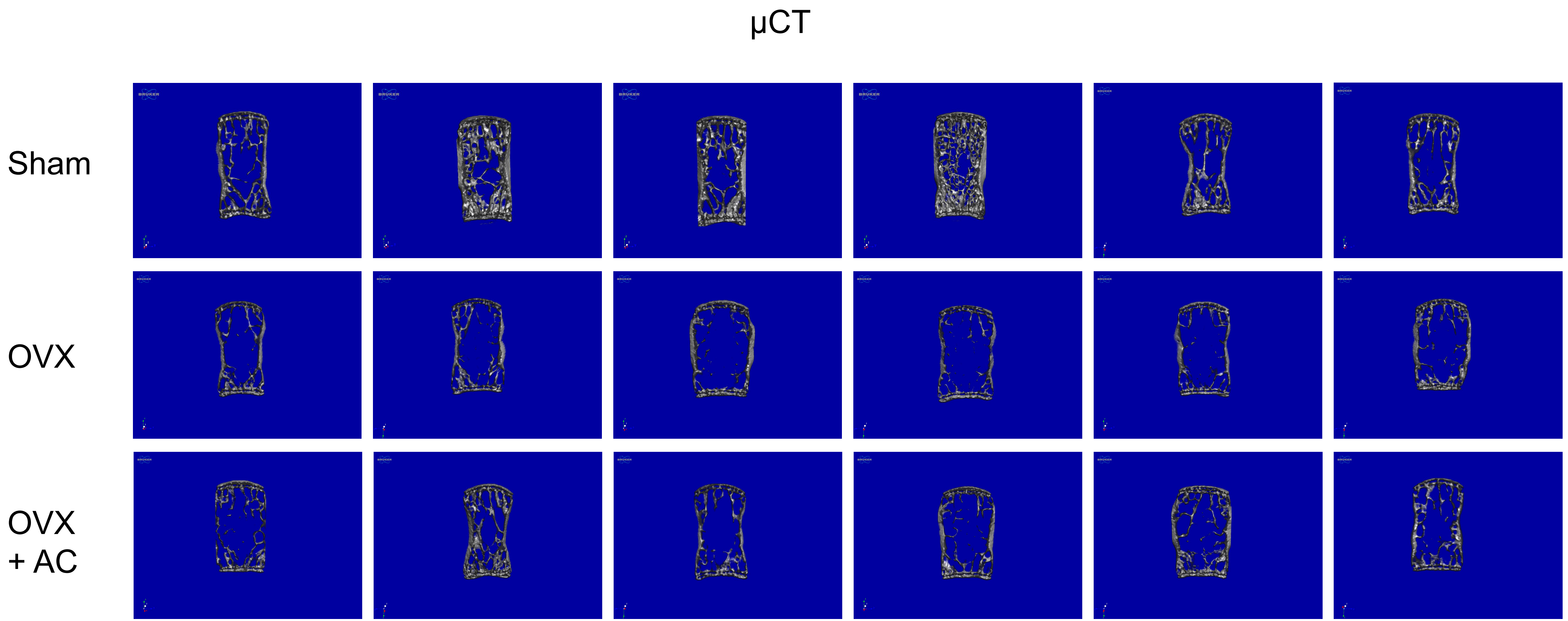

Figure 1B

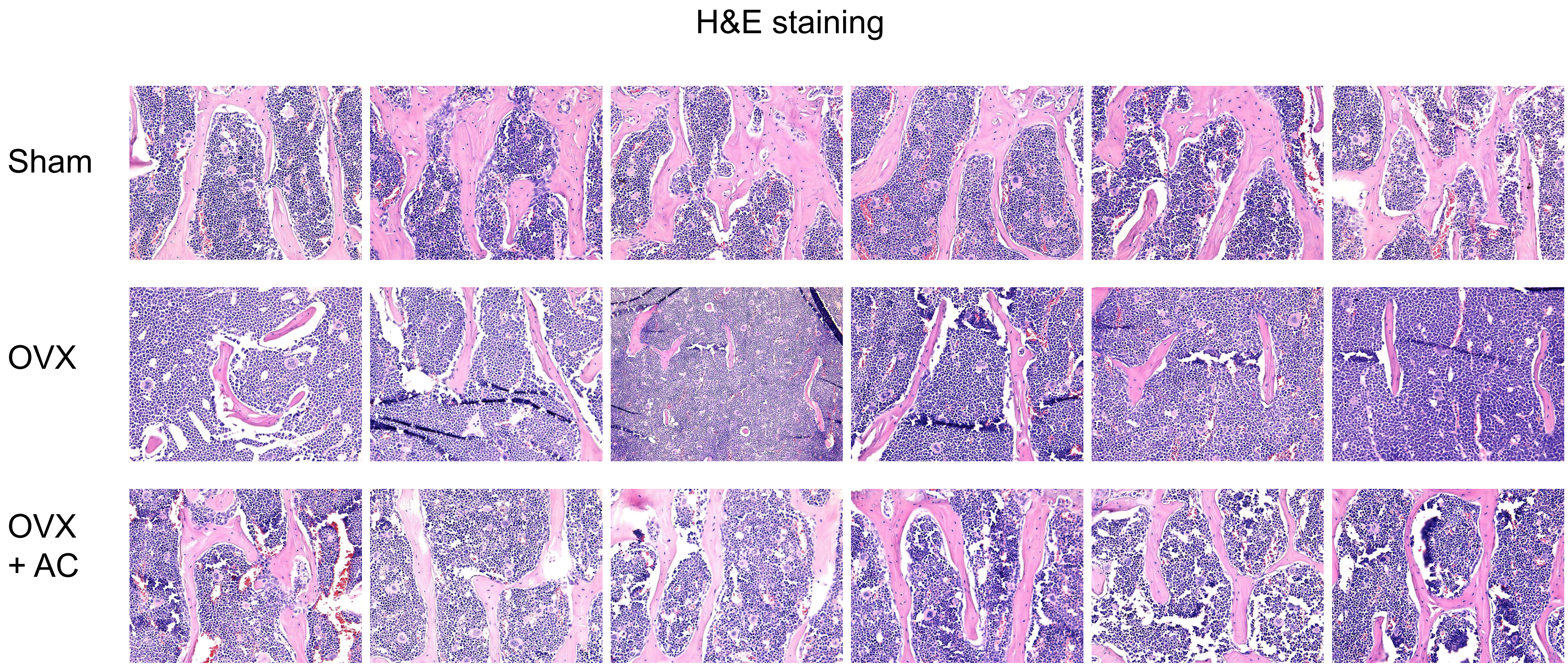

Figure 2D

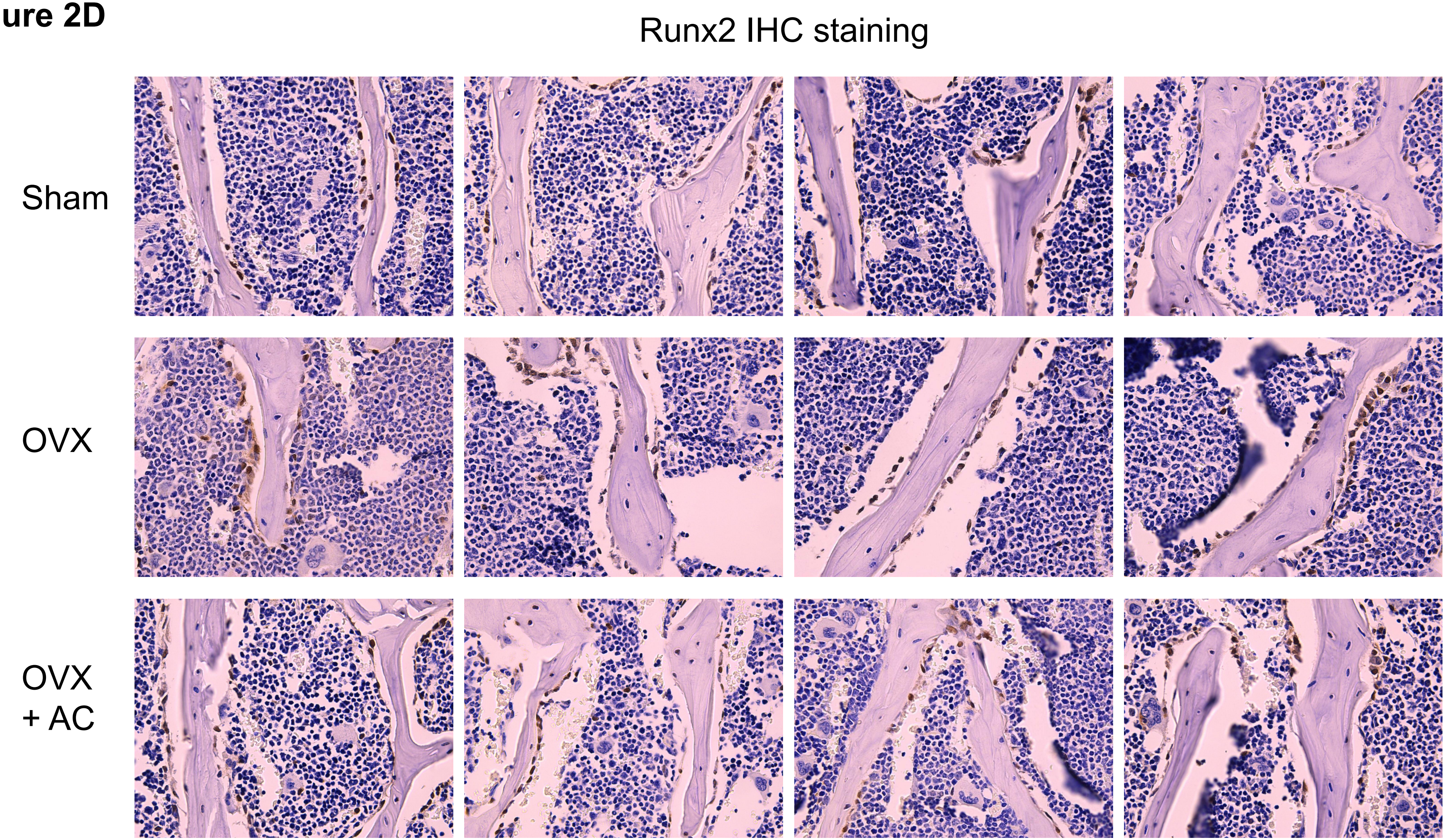

Figure 2E

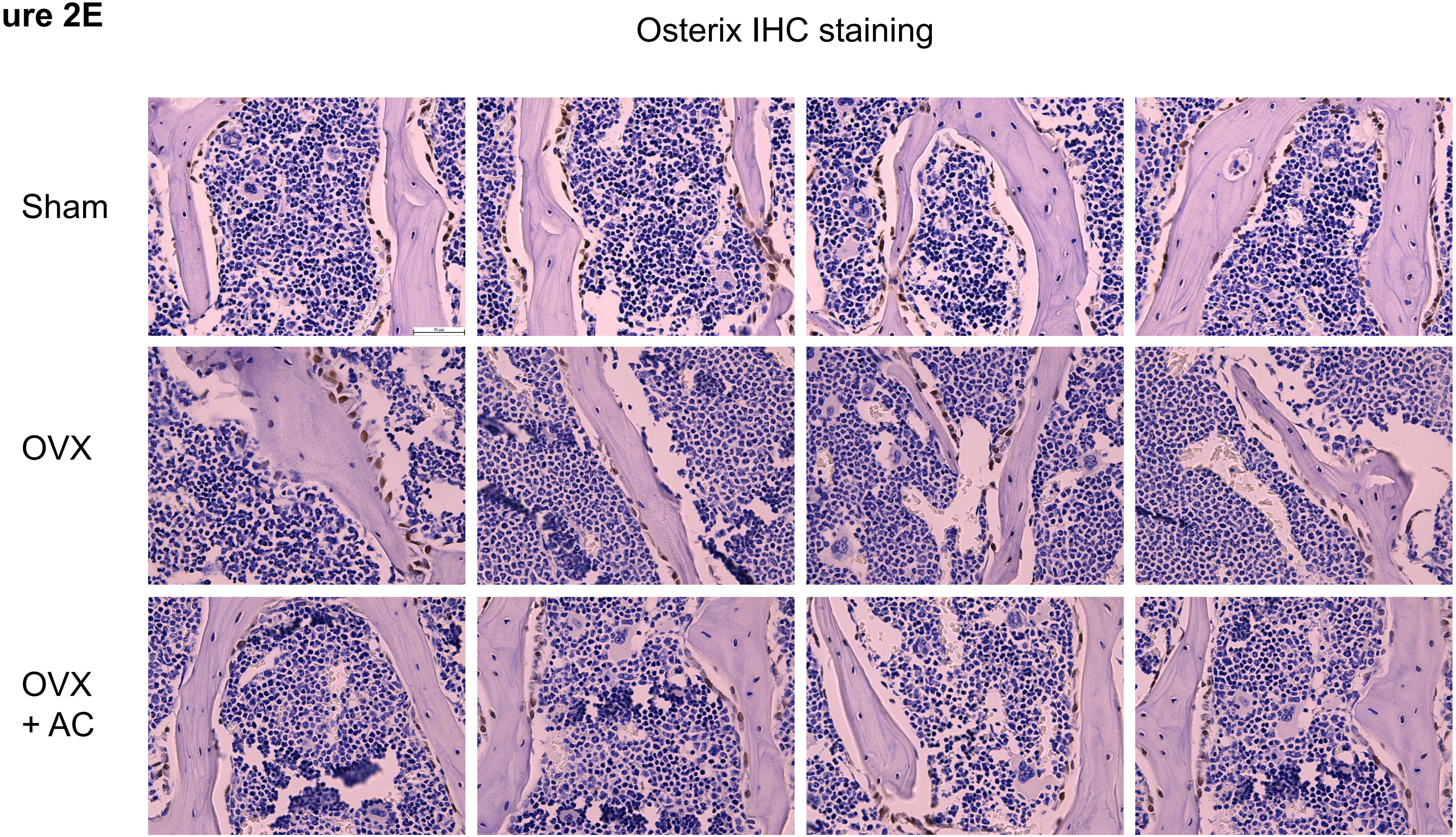

Figure 2F

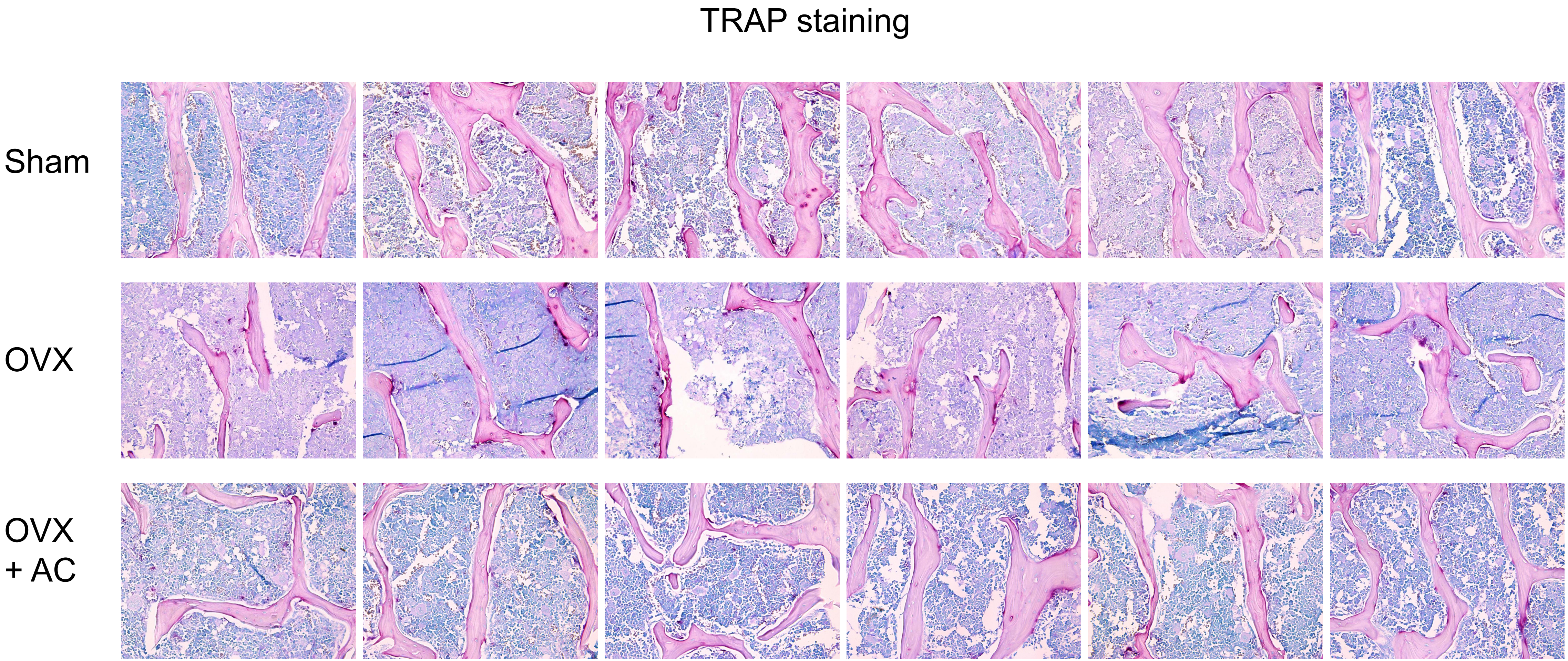

Supplement: Supplementary file 1 [file DataSheet_1.pdf]
