# Supplementary material for: Aconine attenuates osteoclast-mediated bone resorption and ferroptosis to improve osteoporosis via inhibiting NF-κB signaling
Source: Front Endocrinol (Lausanne). 2023 Nov 13;14:1234563. doi: 10.3389/fendo.2023.1234563 (PMC10682992; doi:10.3389/fendo.2023.1234563)

Figure 3A

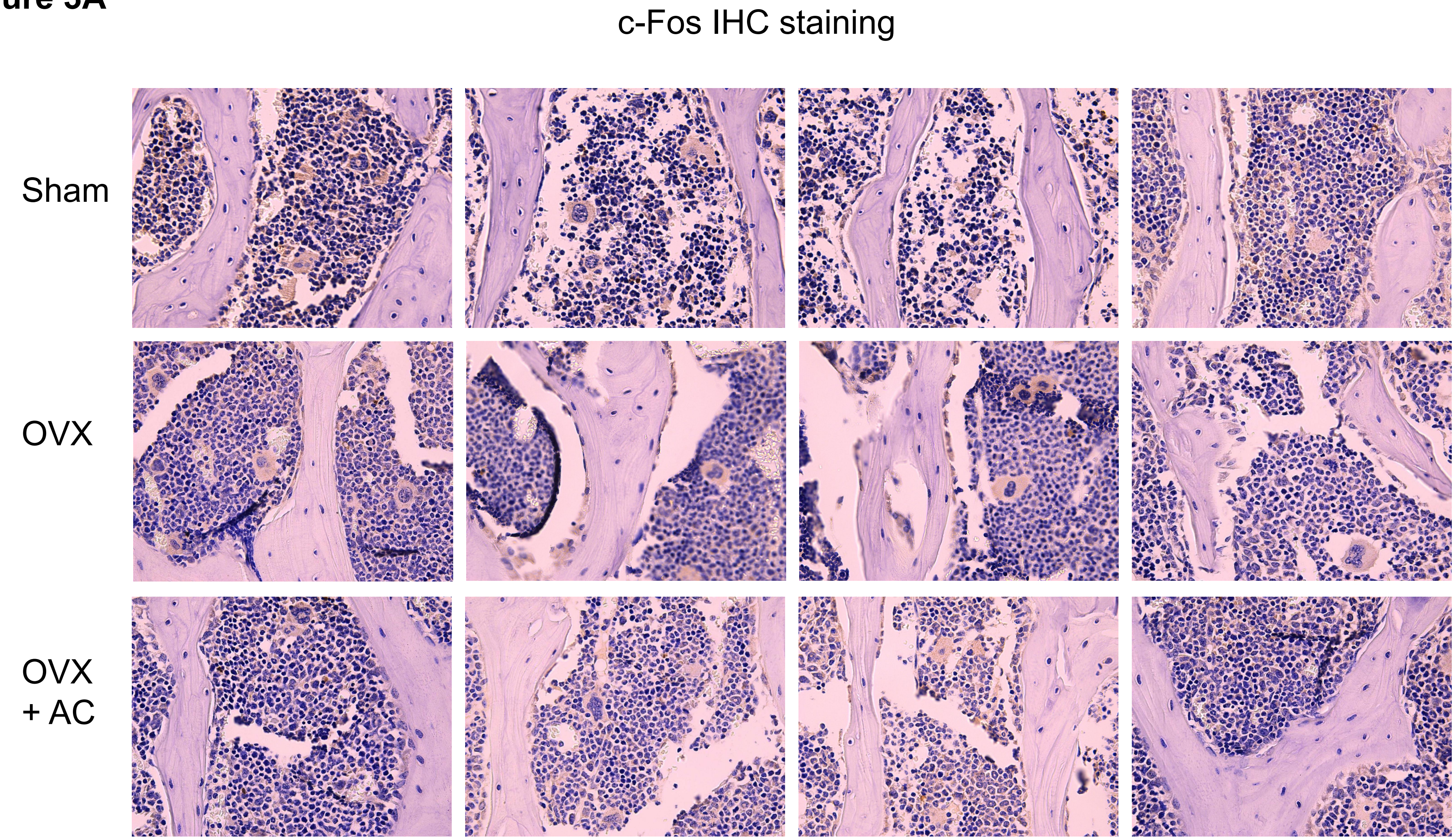

Figure 3C

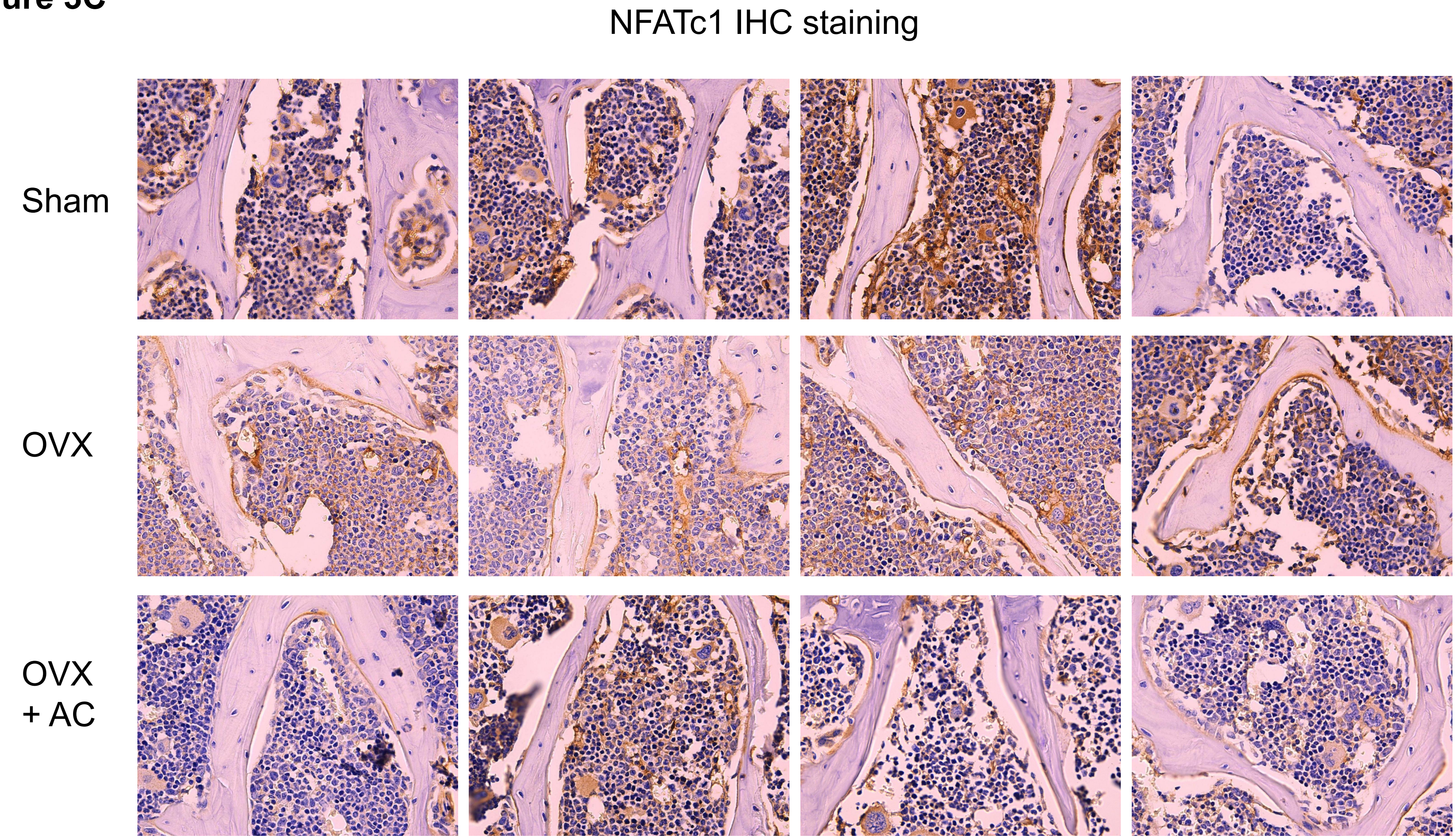

Figure 3G

Cathepsin K IHC staining

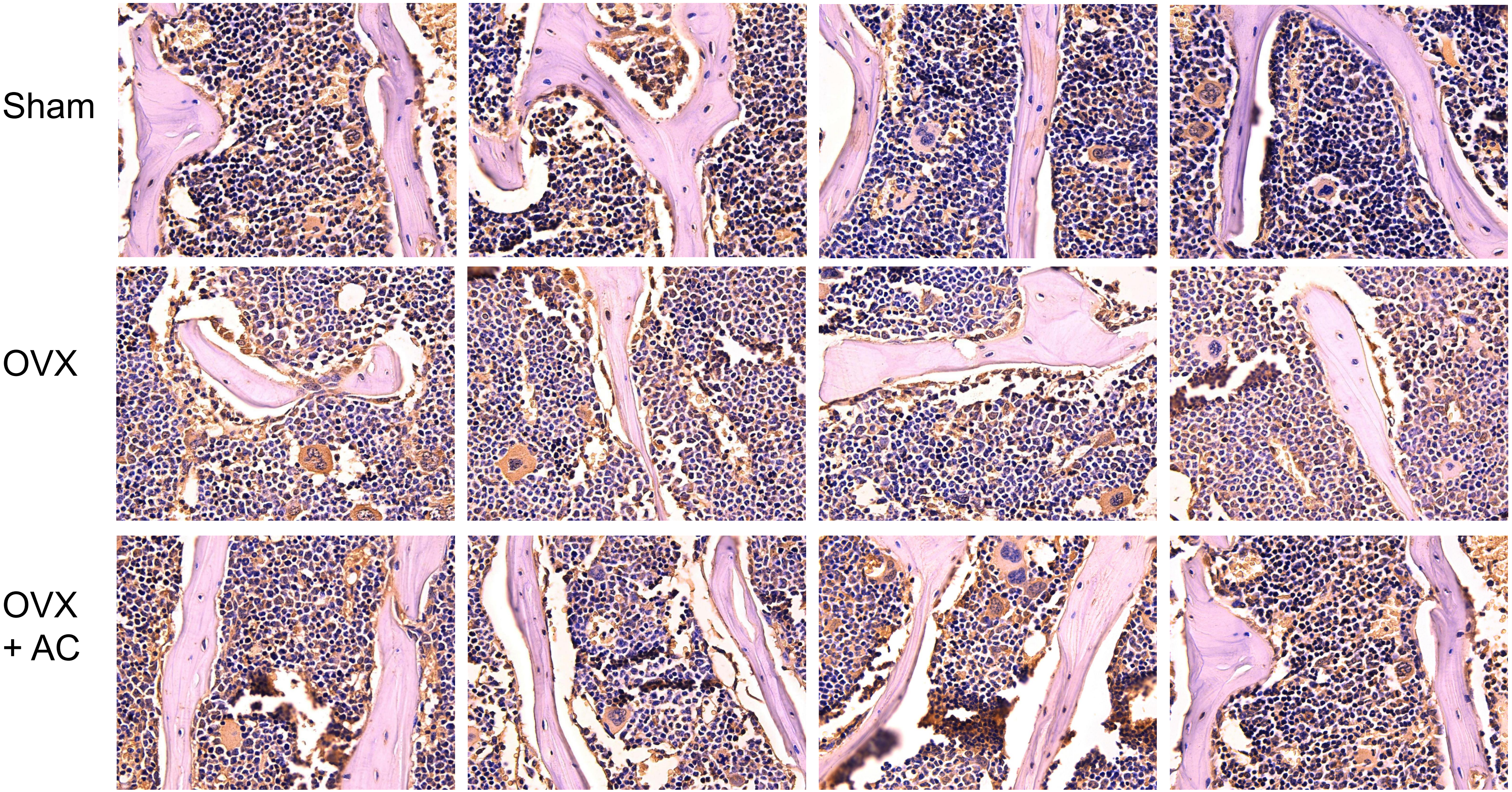

Figure 3E

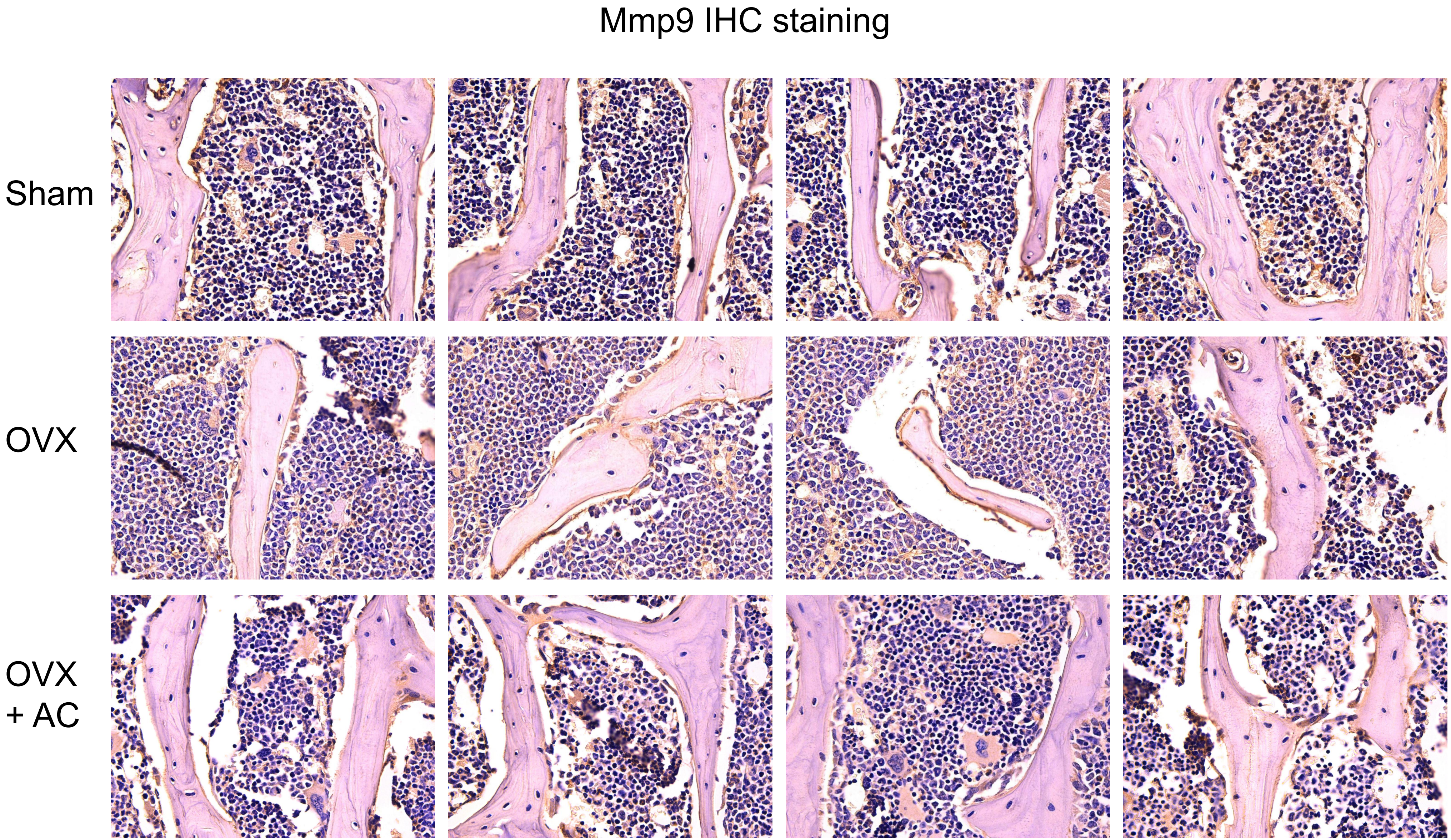

Figure 3I

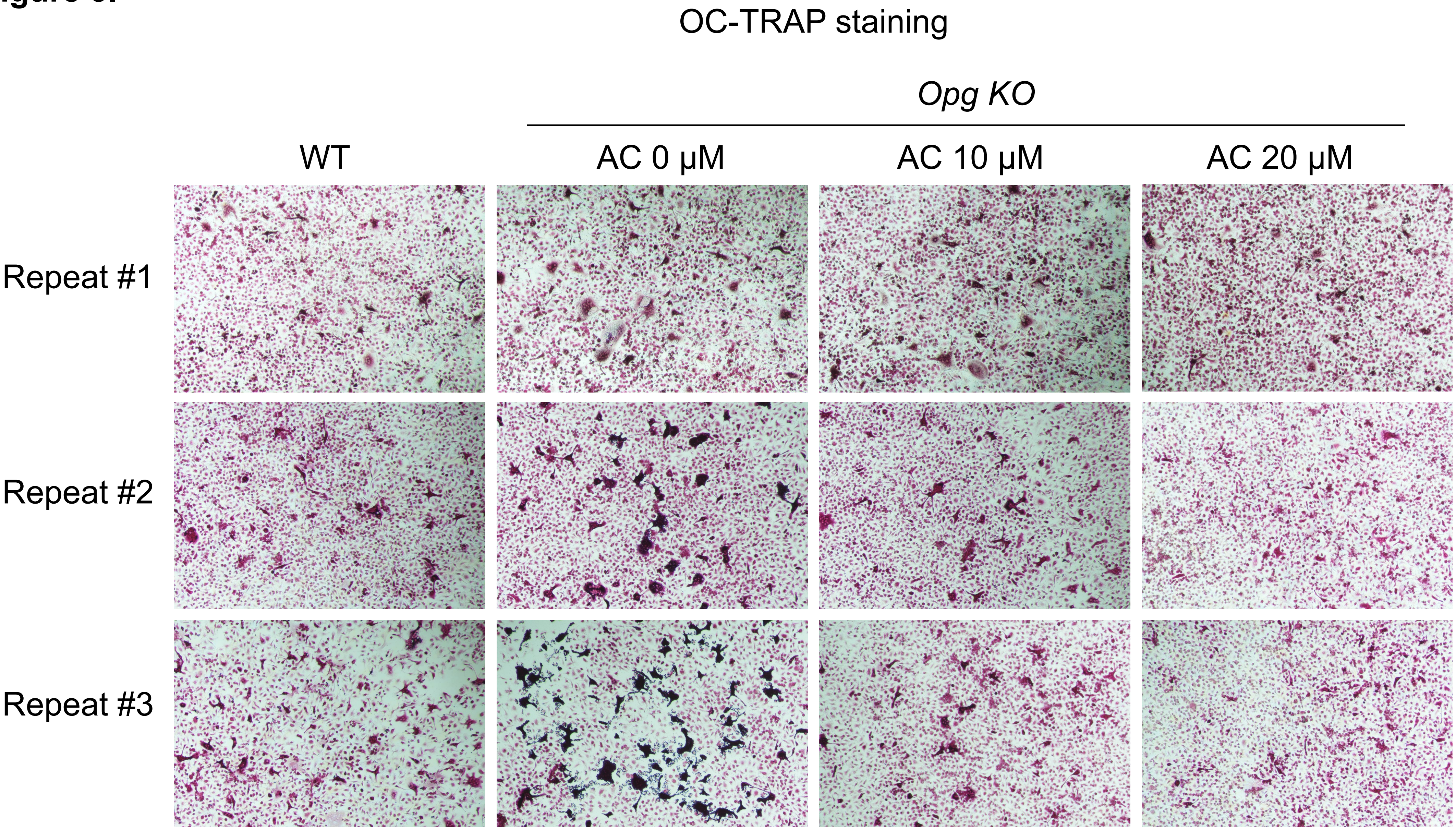

OC-TRAP staining
Opg KO
WT
AC 0 μM
AC 10 μM
AC 20 μM
Repeat #1
Repeat #2
Repeat #3

Figure 4A

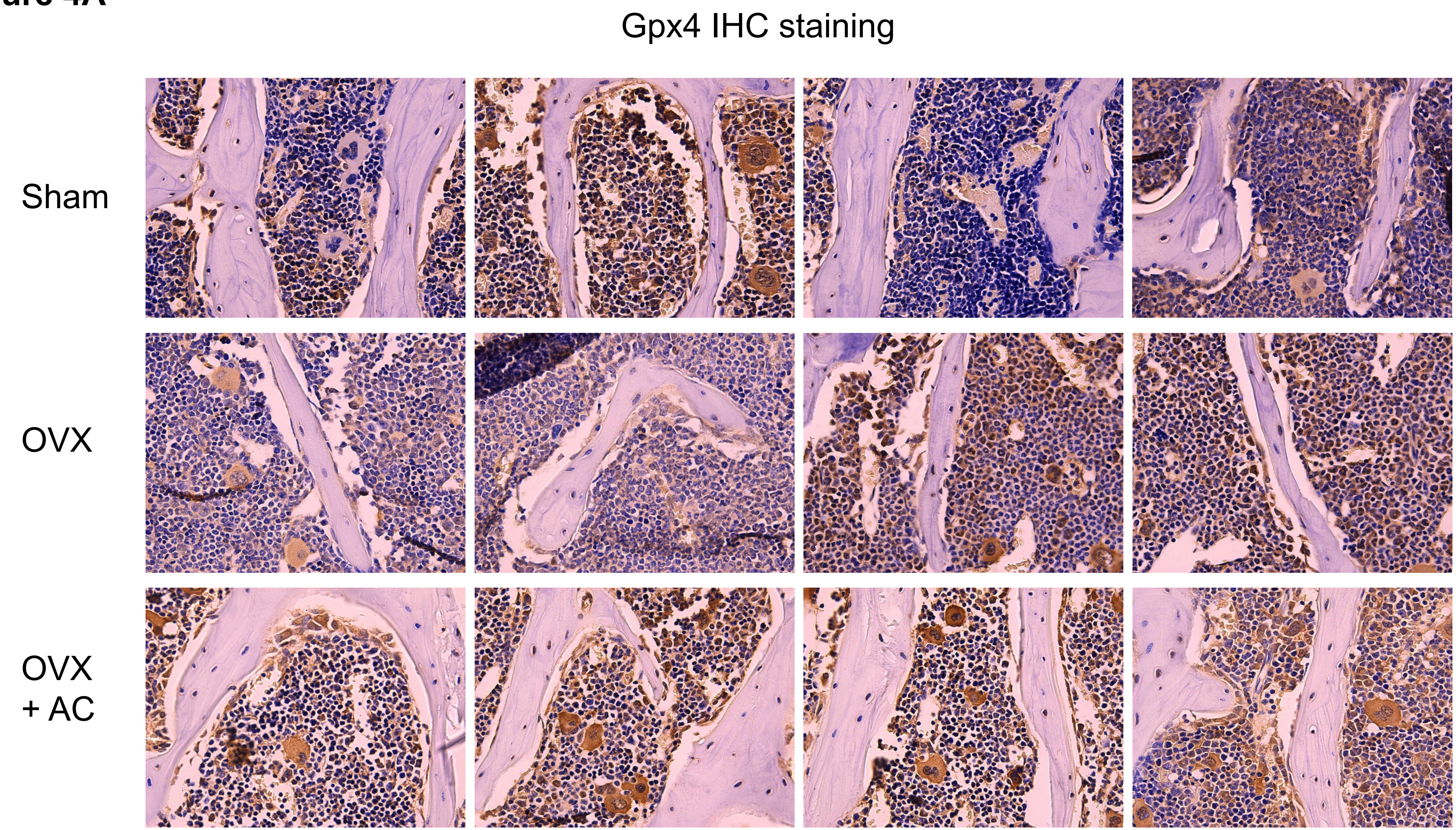

Figure 4C

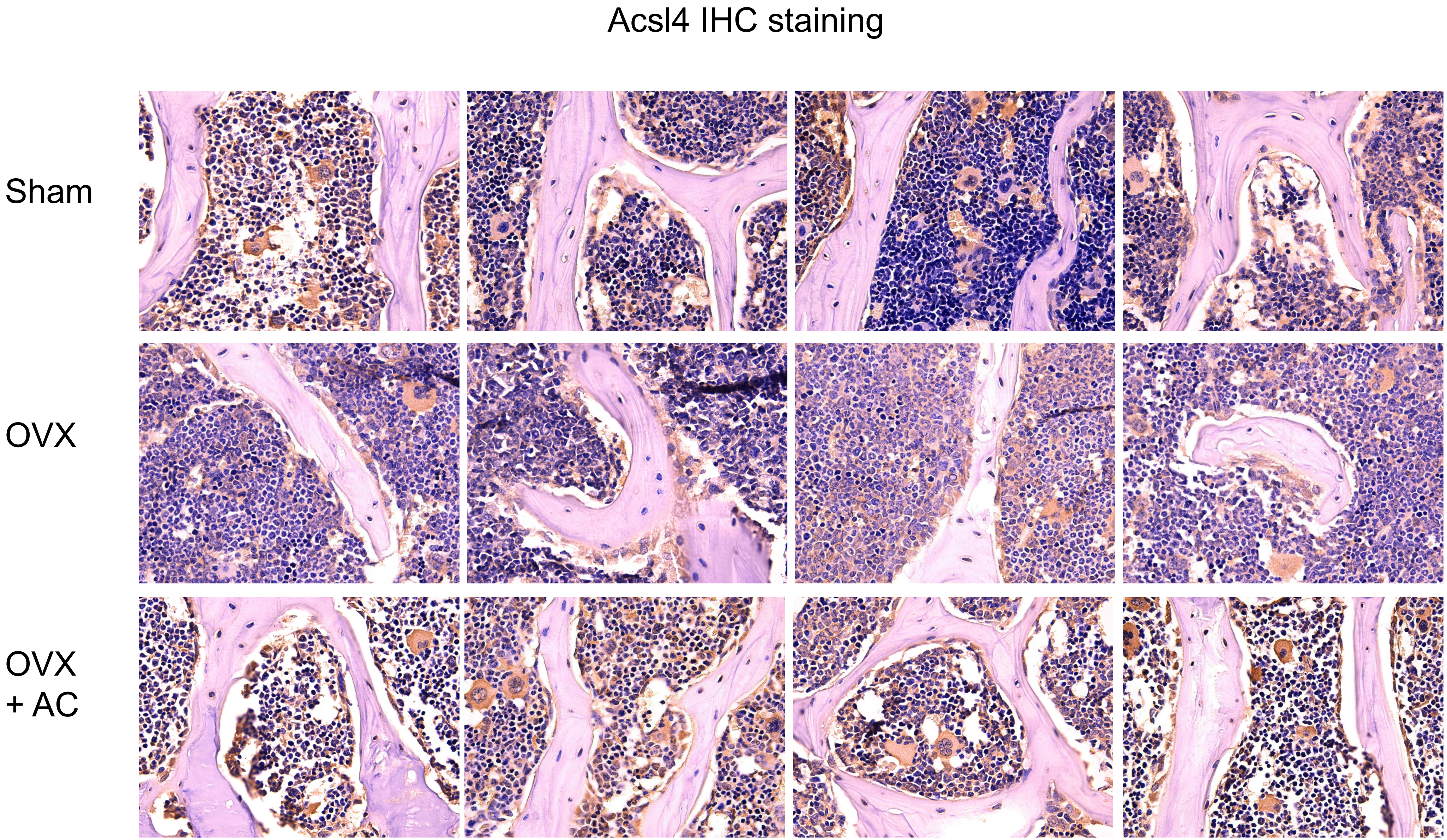

Figure 5A

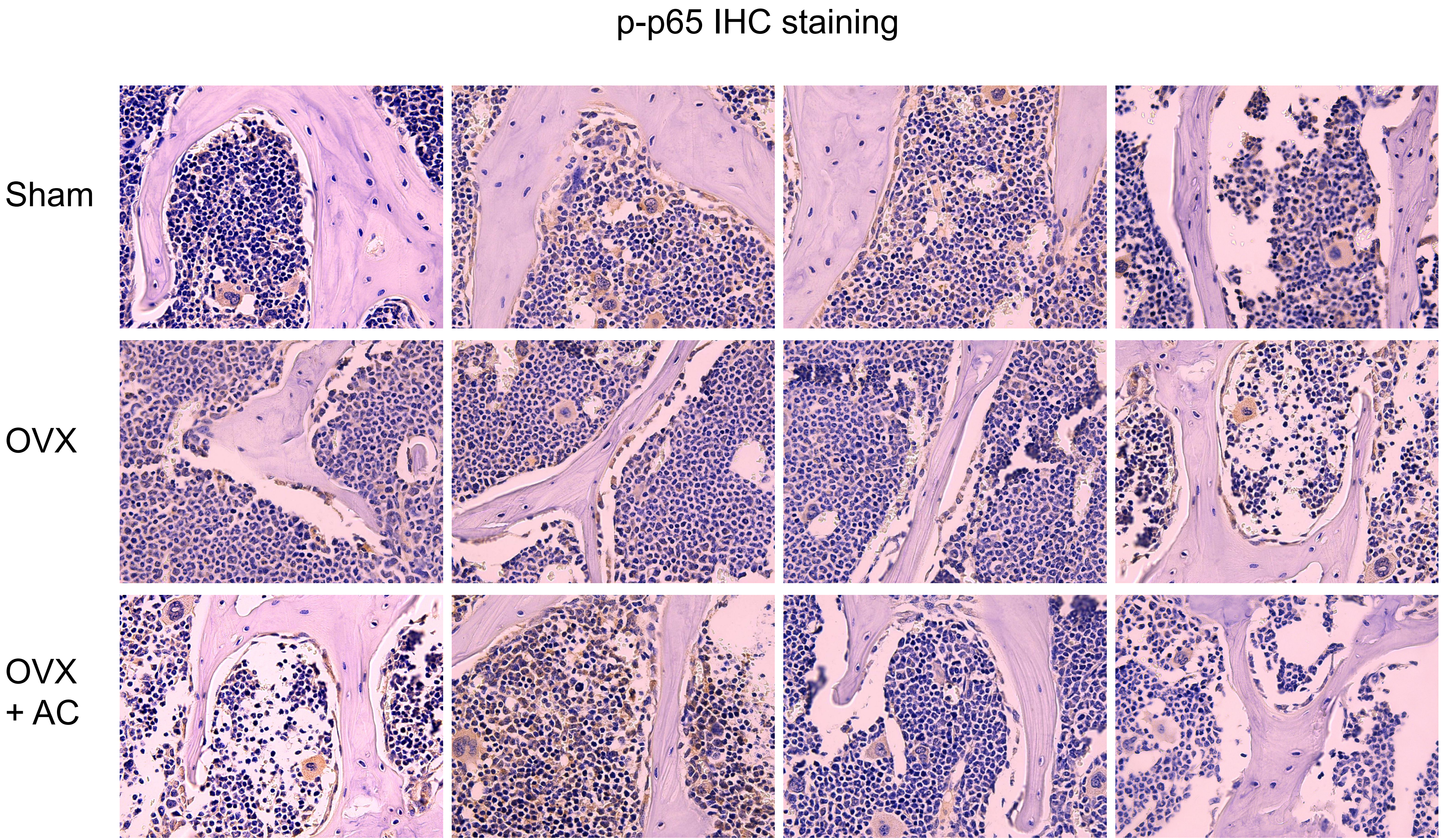

Figure 5C

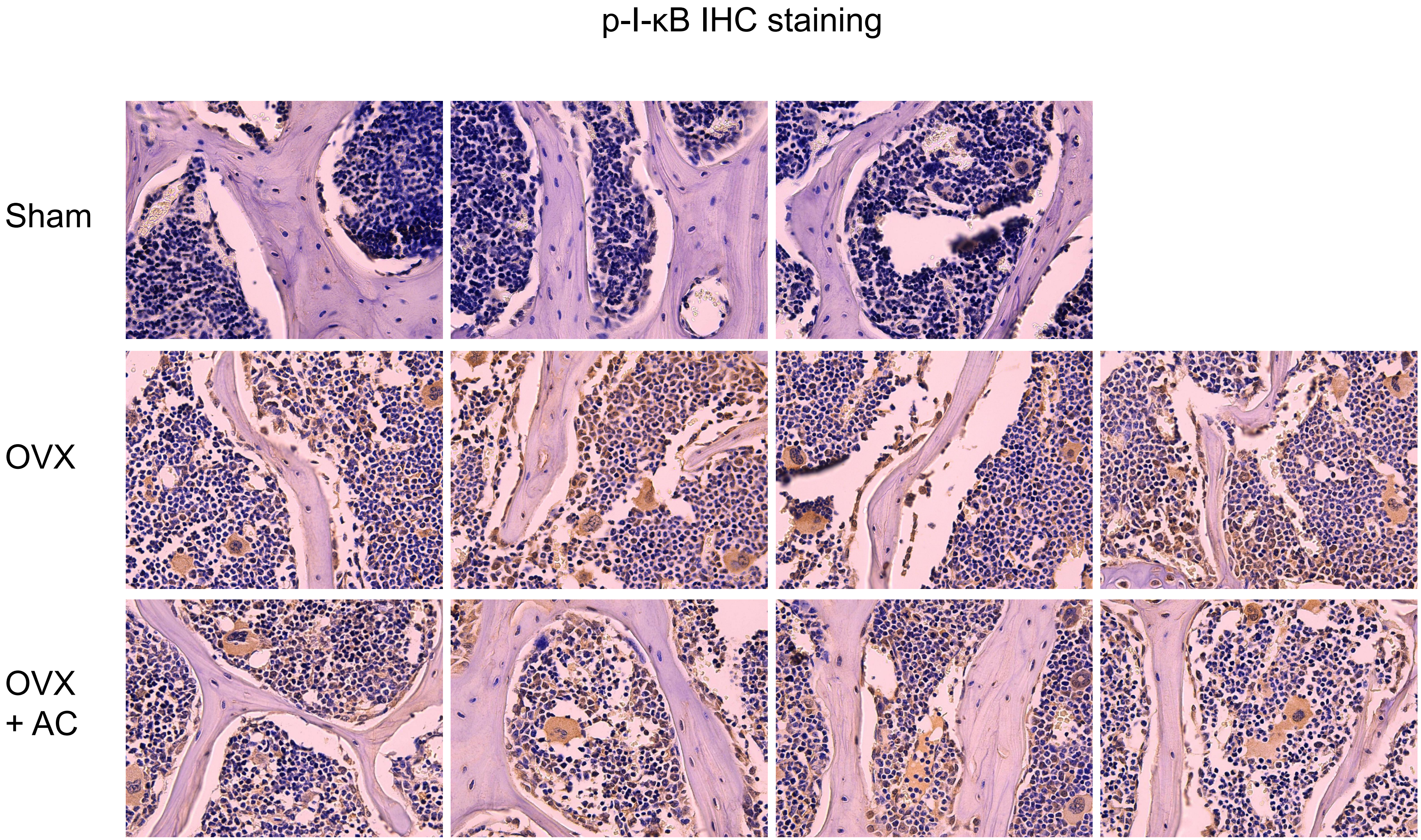

Supplement: Supplementary file 2 [file DataSheet_2.pdf]
