# Supplementary material for: Aconine attenuates osteoclast-mediated bone resorption and ferroptosis to improve osteoporosis via inhibiting NF-κB signaling
Source: Front Endocrinol (Lausanne). 2023 Nov 13;14:1234563. doi: 10.3389/fendo.2023.1234563 (PMC10682992; doi:10.3389/fendo.2023.1234563)
Supplement: Supplementary file 3 [file DataSheet_3.docx]

**Supporting Information**

**Aconine Attenuates Osteoclast-mediated Bone Resorption and Ferroptosis to Improve Osteoporosis via Inhibiting NF-κB Signaling**

Chunchun Xue^1†^, Huan Luo^2†^, Libo Wang^3†^, Qing Deng^1^, Wenyun Kui^1^, Weiwei Da^1^, Lin Chen^1^, Shuang Liu^1^, Yongpeng Xue^1^, Jiafan Yang^1^, Lingxing Li^1^, Wenlan Du^1^, Qi Shi^1^ and Xiaofeng Li^1*^

*^1^Shanghai Municipal Hospital of Traditional Chinese Medicine, Shanghai University of Traditional Chinese Medicine, Shanghai, China, ^2^Department of Pharmacy, the Second Affiliated Hospital, Zhejiang University School of Medicine, Hangzhou, China, ^3^Longhua Hospital, Shanghai University of Traditional Chinese Medicine, Shanghai, China*

**Materials and methods**

**Osteoblastic Determination and Mineralization Assessment**

Osteoblastic MC3T3-E1 cell line was used to evaluate the effects of AC on osteoblast differentiation. The MC3T3-E1 cell line were seeded in a 24-well plate containing 10% fetal bovine serum in α-MEM medium overnight, the next day 10 mM β-glycerophosphate (G5422, Sigma-Aldrich), 50 μg/ml l-ascorbic acid (A8960, Sigma-Aldrich), 10 nM dexamethasone (D4902, Sigma-Aldrich) were added to induce osteogenic differentiation in the presence or absence of 20 μM AC, and the culture was changed every 2 days. After 21 days of differentiation, the differentiated osteoblasts were fixed with 4% paraformaldehyde for 10 minutes. Alkaline phosphatase (ALP) staining was then performed with BCIP/NBT solution (34042, Thermo). Mineralization ability was stained with 1% Alizarin Red-s (AR-S) solution (G1452, Solarbio).

**CCK-8 assay**

Cell viability was assessed by a CCK-8 assay kit (absin, #abs50003). The mouse leukemic monocyte/macrophage cell line RAW 264.7 cells were seeded in 96-well plates at a density of 5000 per well and incubated culture medium overnight. Different concentrations of AC (0, 10, or 20 μM) were added to each well and then incubated for another 24h. Then CCK-8 working solution (10 μl/well) was added to each well for 2 hours at 37°C. The effect of AC on cells was measured by absorbance at 450 nm using a a microplate reader (BioTek, CA, USA).

**Figures**


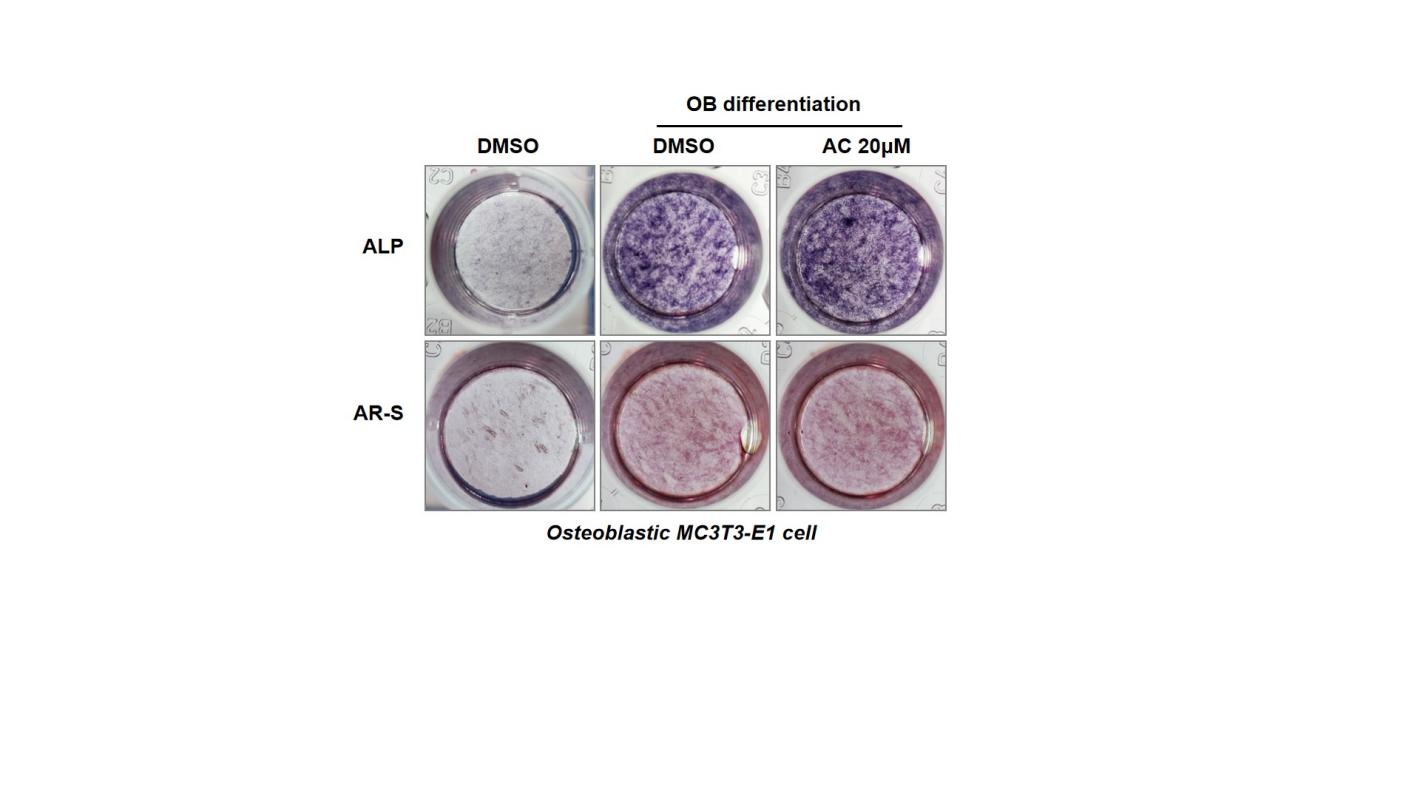


**Fig. S1.** Effect of AC on osteoblast differentiation in each group.

**Fig. S2.** Effects of AC on the cytotoxicity of RAW264.7 cells.
