# Supplementary material for: Aconine attenuates osteoclast-mediated bone resorption and ferroptosis to improve osteoporosis via inhibiting NF-κB signaling
Source: Front Endocrinol (Lausanne). 2023 Nov 13;14:1234563. doi: 10.3389/fendo.2023.1234563 (PMC10682992; doi:10.3389/fendo.2023.1234563)
Supplement: Supplementary file 4 [file Table_1.docx]

Supplementary Table 1 : Real-time Polymerase Chain Reaction (PCR) Primers

| Genes | Forward primer (5′ to 3′) | Reverse Primer (5′ to 3′) |
| --- | --- | --- |
| *c-fos* | CGGGTTTCAACGCCGACTA | TTGGCACTAGAGACGGACAGA |
| *Nfatc1* | GGAGCGGAGAAACTTTGCG | GTGACACTAGGGGACACATAACT |
| *Cathepsin K* | CAGCAGAACGGAGGCATTGA | CTTTGCCGTGGCGTTATACATACA |
| *Mmp9* | CCATGCACTGGGCTTAGATCA | GGCCTTGGGTCAGGCTTAGA |
| *β-actin* | TGTTACCAACTGGGACGACGACA | CTGGGTCATCTTTTCACGGT |
