# Supplementary material for: Aconine attenuates osteoclast-mediated bone resorption and ferroptosis to improve osteoporosis via inhibiting NF-κB signaling
Source: Front Endocrinol (Lausanne). 2023 Nov 13;14:1234563. doi: 10.3389/fendo.2023.1234563 (PMC10682992; doi:10.3389/fendo.2023.1234563)
Supplement: Supplementary file 6 [file Presentation_1.pptx]

## Slide 1
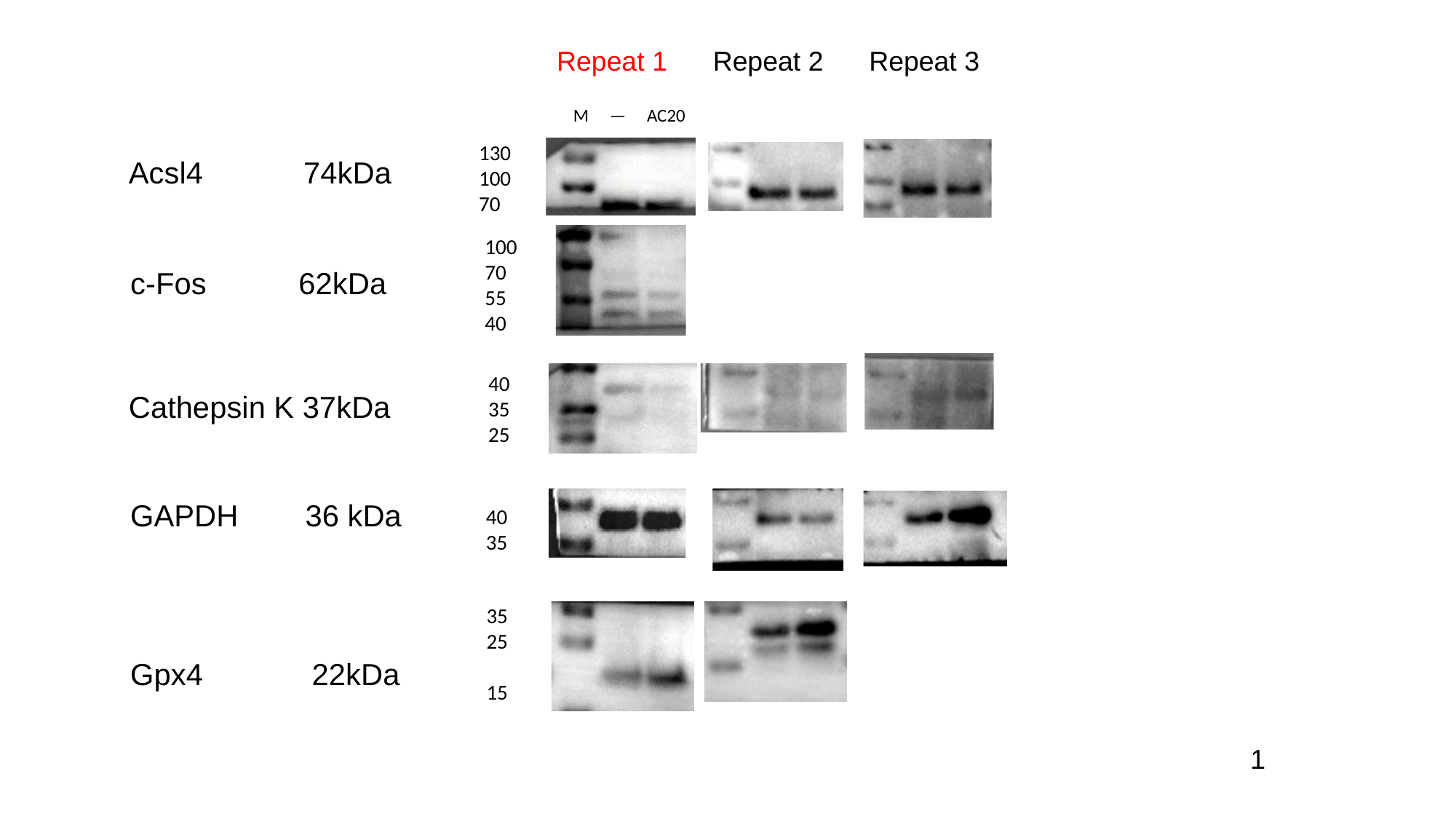

Repeat 1 Repeat 2 Repeat 3
M — AC20
130
100
70
Acsl4 74kDa
100
70
55
40
c-Fos 62kDa
40
35
25
Cathepsin K 37kDa
GAPDH 36 kDa
40
35
35
25
15
Gpx4 22kDa
1

## Slide 2
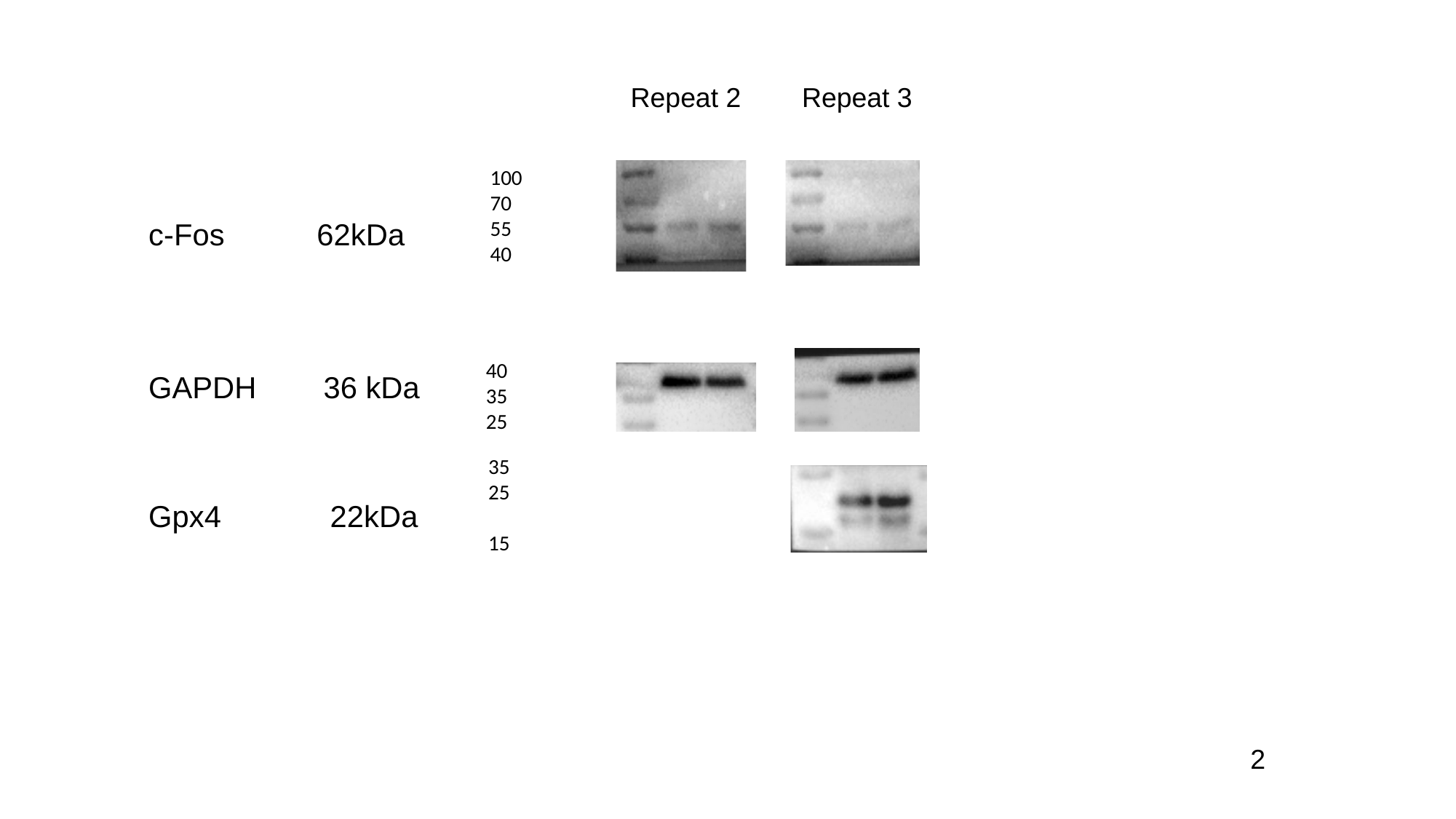

Repeat 2 Repeat 3
100
70
55
40
c-Fos 62kDa
40
35
25
GAPDH 36 kDa
35
25
15
Gpx4 22kDa
2

## Slide 3
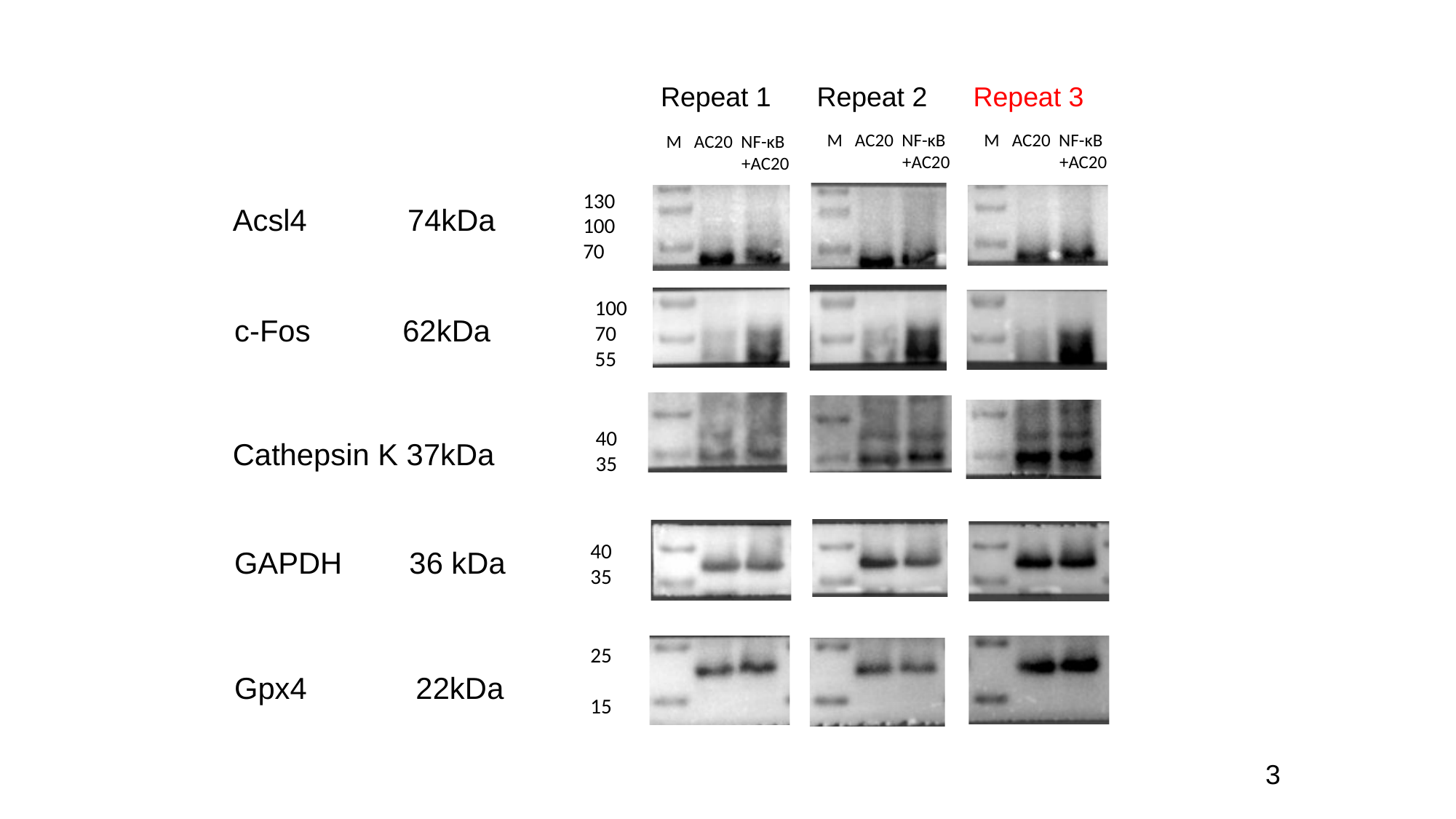

Repeat 1 Repeat 2 Repeat 3
M AC20 NF-κB
 +AC20
M AC20 NF-κB
 +AC20
M AC20 NF-κB
 +AC20
130
100
70
Acsl4 74kDa
100
70
55
c-Fos 62kDa
40
35
Cathepsin K 37kDa
40
35
GAPDH 36 kDa
25
15
Gpx4 22kDa
3
